# Regulation of the *Yersinia pseudotuberculosis* Type III Secretion System by the CpxAR Two‐Component System

**DOI:** 10.1111/mmi.70076

**Published:** 2026-05-24

**Authors:** Karen Hug, Erin Mettert, Melissa Estrada Escobar, Cristina Vargas Vazquez, David Balderas, Sarvind Tripathi, Laya Ashley, Patricia J. Kiley, Victoria Auerbuch

**Affiliations:** ^1^ Department of Microbiology & Environmental Toxicology University of California Santa Cruz Santa Cruz California USA; ^2^ Department of Biomolecular Chemistry University of Wisconsin—Madison Madison Wisconsin USA; ^3^ Department of Chemistry & Biochemistry University of California Santa Cruz Santa Cruz California USA

## Abstract

The type III secretion system (T3SS) is a cell envelope‐spanning injectisome found in many Gram‐negative pathogens. Expression of the T3SS is controlled by extracellular signals such as cell envelope stress. The CpxAR two‐component system negatively regulates the *Yersinia pseudotuberculosis* Ysc T3SS, although the mechanism remains unclear. As expected, we found that mutants with constitutive CpxR activity (∆*cpxA* and *cpxR* ^D51E^) led to decreased expression of the Ysc T3SS and its master regulator LcrF. CpxR did not bind to the regulatory regions of *lcrF*, suggesting an indirect mechanism of regulation. Transcriptome analysis showed that 101 genes were upregulated and 77 genes downregulated in both the ∆*cpxA* and *cpxR* ^D51E^ strains compared to wildtype, including seven genes known to modulate transcription. Individual deletion of these seven regulatory factors did not identify any single gene responsible for CpxR‐dependent repression of LcrF, suggesting that CpxAR may act through multiple pathways to regulate the Ysc T3SS. However, this analysis led us to examine the role of the osmolarity sensing two‐component regulatory system OmpR/EnvZ. Deletion of *ompR* led to an increase in LcrF and T3SS expression. These results suggest more complex regulation of the Ysc T3SS by two‐component regulatory systems than previously appreciated.

## Graphical abstract

Host tissue cues including temperature, oxygen, and iron availability regulate expression of the *Yersinia* type III secretion system (T3SS) master regulator LcrF. We found that CpxR inhibits LcrF through an indirect, multi‐factorial mechanism and identified the osmolarity‐sensing OmpR/EnvZ system as an additional regulator of LcrF, highlighting integrated environmental control of virulence gene expression.

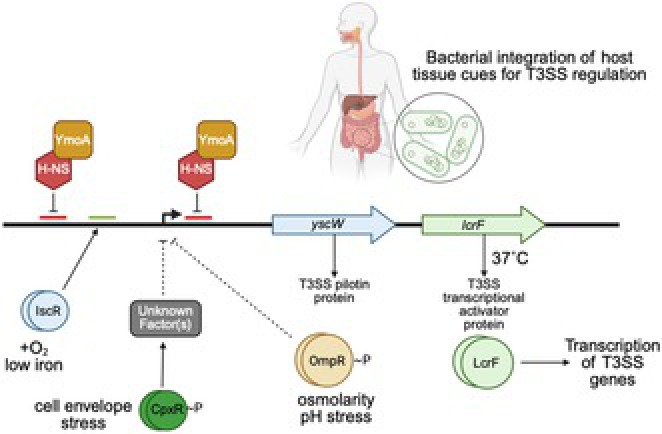

## 1 Introduction

The CpxAR cell envelope stress response system is a two‐component signaling system (TCS) in Gram‐negative bacteria that responds to a variety of extracellular stresses to maintain bacterial cell envelope homeostasis (Raffa and Raivio 2002). It is composed of the inner membrane sensor histidine kinase, CpxA, and the cytoplasmic response regulator and transcription factor CpxR. Several different signals have been shown to activate the CpxAR system, likely through triggering lipoprotein mislocalization or periplasmic protein misfolding, both of which are sensed by CpxA (Danese and Silhavy 1998; Delhaye et al. 2019; Isaac et al. 2005). CpxA integrates stress signals either through direct interaction with the mislocalized lipoprotein sensor NlpE (Delhaye et al. 2019; Marotta et al. 2023) or titration of the CpxP periplasmic chaperone by misfolded periplasmic proteins, which normally represses CpxA autophosphorylation under homeostatic conditions (Danese and Silhavy 1998; Isaac et al. 2005; Raivio et al. 1999). Upon sensing one of these signals, CpxA uses ATP to autophosphorylate on the cytoplasmic side of the inner membrane, followed by transferring the phosphoryl group to monomeric CpxR (Mechaly et al. 2014; Thanikkal et al. 2012). Phosphorylation of CpxR results in a conformational change that allows it to dimerize and bind to DNA to regulate gene expression and combat cell envelope stress (Thanikkal et al. 2012). Deletion of *cpxA* leads to constitutive CpxR activation because CpxA acts as both a kinase and a phosphatase for CpxR (Raivio et al. 1999), and acetyl phosphate can serve as a nonspecific phosphodonor to CpxR (Lima et al. 2016; Wolfe et al. 2008). While the primary purpose of this system is to respond to threats against cell envelope integrity, the CpxAR TCS has been implicated in regulation of a wide range of cellular processes, including the regulation of virulence factors such as the type III secretion system (T3SS) in *Yersinia pseudotuberculosis* (Carlsson et al. 2007a; Liu et al. 2012).

The T3SS is an essential virulence factor found in many Gram‐negative human pathogens. It is comprised of approximately 25 proteins assembled into a needle‐like injectisome that extends from the cytoplasm through the bacterial cell envelope and culminates in an extracellular needle used to translocate effector proteins into the host cytoplasm, resulting in manipulation of host defenses (Deng et al. 2017). Many pathogens require one or more T3SSs to colonize host tissues. However, expression of the T3SS is thought to be metabolically burdensome, and T3SS activity is associated with bacterial growth inhibition in several pathogens, including *Yersinia*, *Salmonella*, and *Shigella* species (Ben‐Gurion and Shafferman 1981; Carter et al. 1980; Fowler et al. 2009; Kupferberg and Higuchi 1958; Sasakawa et al. 1986; Sturm et al. 2011). Therefore, expression of the T3SS is strictly regulated in response to different environmental signals (Bajunaid et al. 2020; Balderas et al. 2022; De Nisco et al. 2018; de Pina et al. 2021; Lou et al. 2019; Schwiesow et al. 2015). For example, in the facultative pathogen *Yersinia pseudotuberculosis* , the T3SS master regulator LcrF is known to be negatively regulated by H‐NS, a repressor of horizontally‐acquired genes, and its co‐repressor YmoA (Balderas et al. 2022; Böhme et al. 2012; Navarre et al. 2006). LcrF is encoded on the *yscW‐lcrF* operon, which also encodes the YscW T3SS pilotin protein and is controlled by a well‐characterized promoter (Schwiesow et al. 2015). Expression of LcrF is induced by the iron–sulfur cluster regulator IscR in response to low iron or aerobic conditions, and this regulation is critical for *Y. pseudotuberculosis* virulence in a mouse infection model (Balderas et al. 2022; Hooker‐Romero et al. 2019; Miller et al. 2014).

In addition to H‐NS/YmoA and IscR, CpxR has been suggested to directly regulate LcrF (Liu et al. 2012). A *Y. pseudotuberculosis* ∆*cpxA* mutant was shown to have high levels of phosphorylated CpxR and decreased levels of Yop secretion (Carlsson et al. 2007a). Purified CpxR~*P* was reported to bind the *yscW‐lcrF* intergenic region (Liu et al. 2012). However, as no additional promoter mapping to the *yscW‐lcrF* intergenic region has been detected (Nuss et al. 2015), the significance of CpxR binding to the *yscW‐lcrF* intergenic region remained unclear. In this study, we find that mutations resulting in constitutive activation of CpxR in *Y. pseudotuberculosis* lead to decreased *yscW‐lcrF* promoter activity as well as LcrF and T3SS expression (Huynh et al. 2025). However, our evidence suggests that CpxR regulates LcrF through an indirect mechanism that does not involve factors previously known to control the *yscW‐lcrF* promoter.

## 2 Results

### 2.1 Activation of the Cpx Inner Membrane Stress Response System Results in a CpxR‐Dependent Decrease in T3SS Expression

**FIGURE 1:**
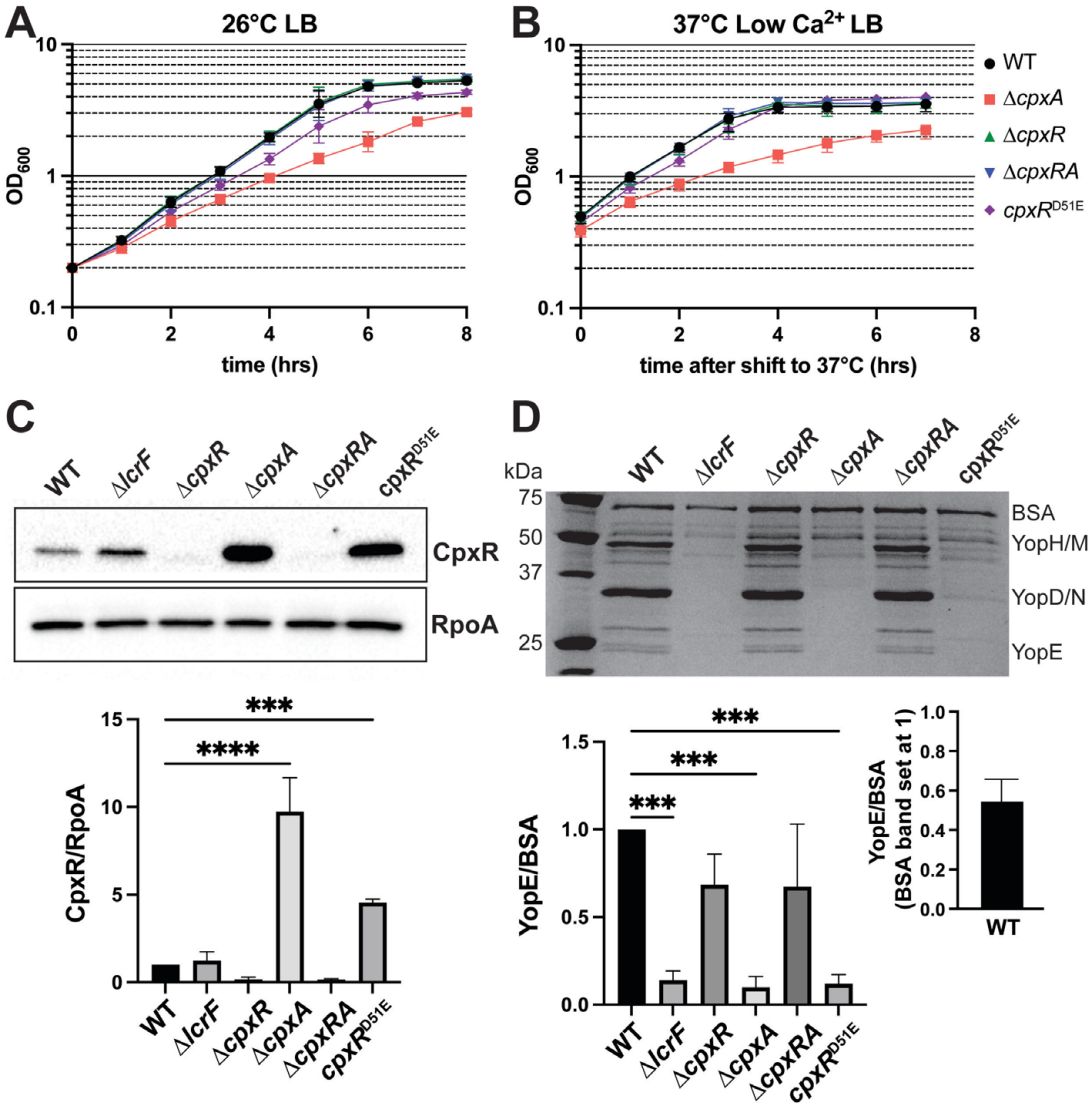
Constitutive activation of CpxR leads to high CpxR protein levels and a defect in T3SS expression. (A) *Y. pseudotuberculosis* strains were grown in LB medium at 26°C for a total of 8 h. OD_600_ was recorded every hour to track growth. Average ± standard deviation (StDev) of three independent experiments is shown. Error bars that are not visible are smaller than the symbol for that data point. (B) The same strains as in (A) were grown in low calcium LB at 26°C for 1.5 h and then shifted to 37°C for a total of 7 h. OD_600_ was recorded before the shift to 37°C and each hour as in (A). Average ± StDev of three independent experiments is shown. (C) Strains were grown in T3SS‐inducing conditions, normalized for cell density, and whole cell lysates collected for western blotting using CpxR‐ and RpoA‐specific antibodies. Densitometry was used to measure the relative amount of CpxR relative to the RpoA control. Average ± StDev of three independent experiments is shown. (D) Secreted proteins were precipitated from the supernatant of the same strains in (C) and visualized on a 12.5% SDS PAGE gel stained with Coomassie Blue. Densitometry was used to measure the amount of YopE relative to the BSA protein precipitation control for each sample. The WT YopE/BSA ratio was set at 1 for each experiment. Average ± StDev of three independent experiments is shown. Inset shows the standard deviation for the WT strain over four independent experiments. *p < 0.05, **p < 0.01, ***p < 0.001, ****p < 0.0001 (one‐way ANOVA with Dunnett comparison).

To establish how the CpxAR TCS regulates the Ysc T3SS in the *Y. pseudotuberculosis* IP2666pIB1 strain, we assessed growth, CpxR protein levels, and secretion of T3SS effector proteins in *Y. pseudotuberculosis* IP2666pIB1 mutants predicted to have high constitutive levels of CpxR activation: ∆*cpxA* and the phosphomimetic mutant *cpxR* ^D51E^ (Thanikkal et al. 2012). In standard growth conditions (26°C, LB), the Δ*cpxA* mutant exhibited a marked growth defect with a log phase doubling 1.6‐fold slower than wildtype (Figure 1A). This growth defect in the ∆*cpxA* mutant could be rescued by deletion of *cpxR* (Figure 1A). While the *cpxR* ^D51E^ mutant also exhibited a growth defect at 26°C, this was not as severe as the ∆*cpxA* strain. In contrast, under T3SS‐inducing conditions (37°C, low calcium LB), only the ∆*cpxA* mutant showed significantly decreased growth compared to wildtype with a log phase doubling 1.7‐fold slower than wildtype (Figure 1B). The same strains were also evaluated for CpxR protein levels (Figure 1C) and T3SS activity (Figure 1D). CpxR is positively autoregulated, and total CpxR levels should reflect the amount of active CpxR (De Wulf et al. 1999; Liu et al. 2011; Raivio et al. 1999). CpxR protein levels in the ∆*cpxA* mutant were ~10‐fold higher than in the wildtype strain whereas the *cpxR* ^D51E^ mutant had ~4.5‐fold increased CpxR levels, suggesting that ∆*cpxA* cells contain higher levels of activated CpxR than the *cpxR* ^D51E^ mutant. Expression of the T3SS effector protein YopE was inversely correlated with CpxR levels, suggesting that CpxR activation leads to repression of the T3SS, consistent with previous results (Carlsson et al. 2007a, 2007b; Liu et al. 2011). The defect in T3SS expression in the Δ*cpxA* mutant was CpxR‐dependent, as T3SS expression was rescued in a Δ*cpxA*/Δ*cpxR* double mutant. Notably, single deletion of *cpxR* did not influence secretion, indicating that the CpxAR system is not activated in wildtype *Yersinia* under standard T3SS‐inducing culture conditions.

### 2.2 Overexpression of the Lipoprotein NlpE Activates CpxR and Represses LcrF Expression

**FIGURE 2:**
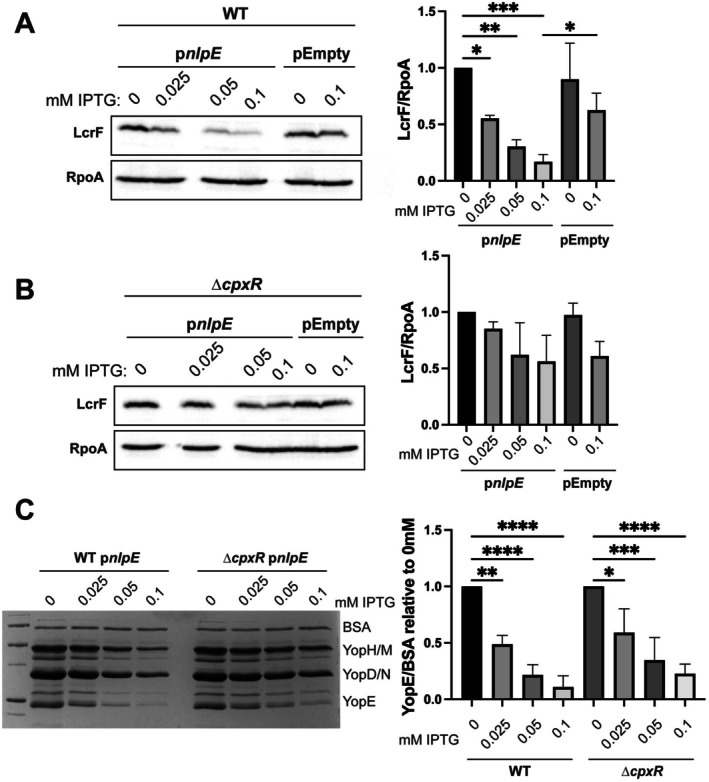
NlpE overproduction, a signal known to activate CpxR, leads to a decrease in LcrF expression. Wildtype and Δ*cpxR* *Y. pseudotuberculosis* strains harboring a pTRC99a‐*nlpE* overexpression vector were grown in T3SS‐inducing conditions in the presence of varying concentrations of IPTG. LcrF levels were assessed via western blot in wildtype (A) and Δ*cpxR* (B) backgrounds. (C) Secreted proteins were precipitated and visualized via Coomassie‐stained SDS‐PAGE. Densitometry was used to measure the amount of YopE relative to the BSA protein precipitation control. Average ± StDev of three independent experiments is shown. *p < 0.05, **p < 0.01, ***p < 0.001, ****p < 0.0001 (one‐way ANOVA with Tukey comparison).

To evaluate the effects of CpxR activation on T3SS expression in response to a known source of envelope stress, we overexpressed the lipoprotein NlpE whose mislocalization is a known signal for the CpxAR system (Danese et al. 1995; Delhaye et al. 2019; Snyder et al. 1995). A previous study showed that overexpressing NlpE in *Y. pseudotuberculosis* leads to an increase in overall CpxR levels as well as an increase in phosphorylated CpxR (Liu et al. 2012). We introduced the plasmid pTRC99a expressing an IPTG‐inducible *nlpE* gene into wildtype and Δ*cpxR* *Y. pseudotuberculosis* strains. Over an IPTG titration ranging from 0 to 0.1 mM IPTG, a clear dose‐dependent reduction in LcrF protein levels was observed in the wildtype strain, and this was dependent on CpxR (Figure 2A,B). However, NlpE overexpression led to a dose‐dependent decrease in Yop secretion in both the wildtype and ∆*cpxR* strains (Figure 2C, empty vector controls Figure S1), suggesting that NlpE overexpression also impacts T3SS activity in a CpxR‐independent manner. This may be due to disruption of T3SS assembly, since it requires proper lipoprotein localization. Taken together, these data suggest that activating CpxR by lipoprotein mislocalization leads to repression of LcrF.

### 2.3 CpxR Activation Decreases LcrF Expression, but Does Not Appear to Directly Repress *lcrF* Transcription

**FIGURE 3:**
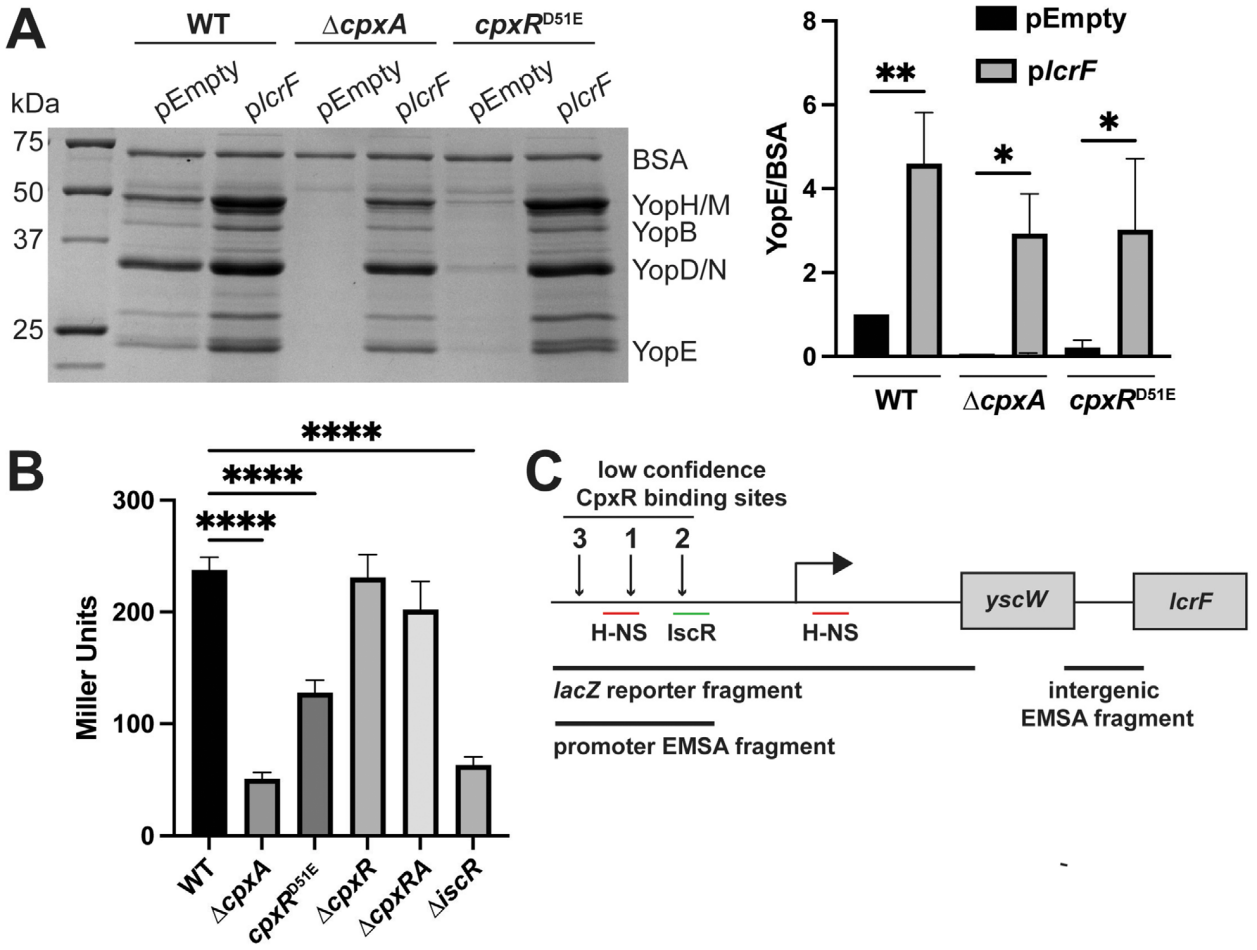
CpxR activation results in lower T3SS expression that can be rescued with overexpression of the T3SS master regulator LcrF. (A) *Y. pseudotuberculosis* strains harboring either pBAD33‐*lcrF* or empty vector were supplemented with 0.4% arabinose to induce expression of LcrF and grown under T3SS‐inducing conditions. Secreted proteins were precipitated from the supernatant and run on SDS‐PAGE for quantification of YopE to determine relative T3SS activity. Densitometry was used to measure the relative amount of YopE relative to the BSA protein precipitation control. Average ± StDev of three independent experiments is shown. (B) *Y. pseudotuberculosis* strains harboring a pFU99a vector encoding the *lacZ* gene driven by the *yscW‐lcrF* promoter were grown in T3SS‐inducing conditions. Samples were normalized to cell density and β‐galactosidase activity of each sample was assessed by addition of ortho‐Nitrophenyl‐β‐galactoside (ONPG). Average ± StDev of three independent experiments is shown. (C) Bioinformatic analysis identified three low confidence CpxR~*P* binding motifs in the *yscW‐lcrF* promoter region. *p < 0.05, **p < 0.01, ***p < 0.001, ****p < 0.0001 (one‐way ANOVA with Tukey comparison).

**FIGURE 4:**
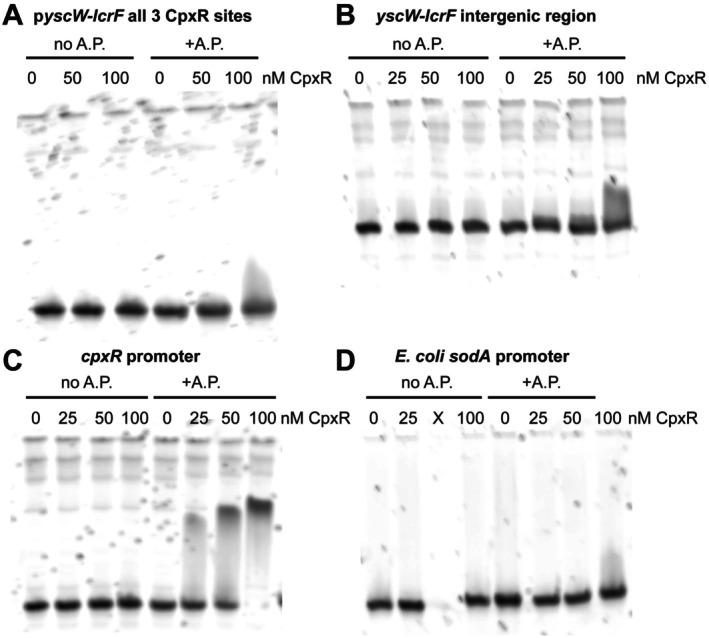
CpxR does not bind to regulatory regions of *yscW‐lcrF*. Fragments of (A) the *yscW‐lcrF* promoter region containing the three putative CpxR~*P* binding motifs (−313 to −74 bp relative to the TSS generated with primers M13R and pPK7179_P*yscW*_R1), (B) the *yscW‐lcrF* intergenic region (+541 to +861), (C) the *cpxR* promoter region (−157 to +16), as well as (D) the *E. coli* *sodA* promoter region (−200 to +40 relative to the TSS) were evaluated for binding by CpxR~*P* in the absence or presence of acetyl phosphate (A.P.) using EMSAs.

We investigated the mechanism by which CpxR represses LcrF. Overexpression of LcrF driven by an arabinose‐inducible promoter recovered the T3SS expression defect of both the Δ*cpxA* and *cpxR* ^*D51E*^ mutant strains, suggesting that CpxR repression of the T3SS occurs either by directly acting on the *yscW‐lcrF* operon or by regulating another factor upstream of LcrF (Figure 3A). Next, we investigated whether the characterized promoter upstream of the *yscW‐lcrF* operon is impacted by CpxR activation using a β‐galactosidase reporter construct containing a fragment −309 to +294 bp relative to the *yscW* transcriptional start site (TSS) (Nuss et al. 2015) (Figure 3B). Promoter activity in both the ∆*cpxA* and *cpxR* ^D51E^ strains was significantly decreased relative to wildtype, indicating that CpxR activation represses the known *yscW*‐*lcrF* promoter. Bioinformatic analysis did not identify any consensus CpxR binding motifs (TTTAC‐N_4‐8_‐TTTAC) (Yamamoto and Ishihama 2006) either in the *yscW‐lcrF* promoter or the *yscW‐lcrF* intergenic region. However, low‐confidence binding sites could be identified. Putative CpxR binding motif 1 is located at position −175 relative to the TSS (*p* = 0.00132), site 2 is located at −120 (*p* = 0.00142), and site 3 at −252 (*p* = 0.00193) (Figure 3C). Binding of purified CpxR~*P* to PCR fragments that included all or a subset of these three sites in the *yscW‐lcrF* promoter region was not detected (Figure 4A, Figure S2) under the same conditions that we observed robust binding of CpxR~*P* to the *cpxR* promoter using 25–100 nM of protein (Figure 4C). Likewise, we could not detect CpxR~P binding to the *yscW‐lcrF* intergenic region (Figure 4B). As expected, we observed no binding of CpxR~P to the *E. coli* *sodA* promoter as a negative control (Figure 4D). Taken together, these data suggest that CpxR does not bind strongly to the *yscW*‐*lcrF* promoter or intergenic region, and instead suggests CpxR regulates LcrF expression through an indirect mechanism.

### 2.4 The *ymoBA* Promoter Region Contains a Bioinformatically Identifiable CpxR Binding Motif and Is Transcriptionally Responsive to CpxR Activation

**FIGURE 5:**
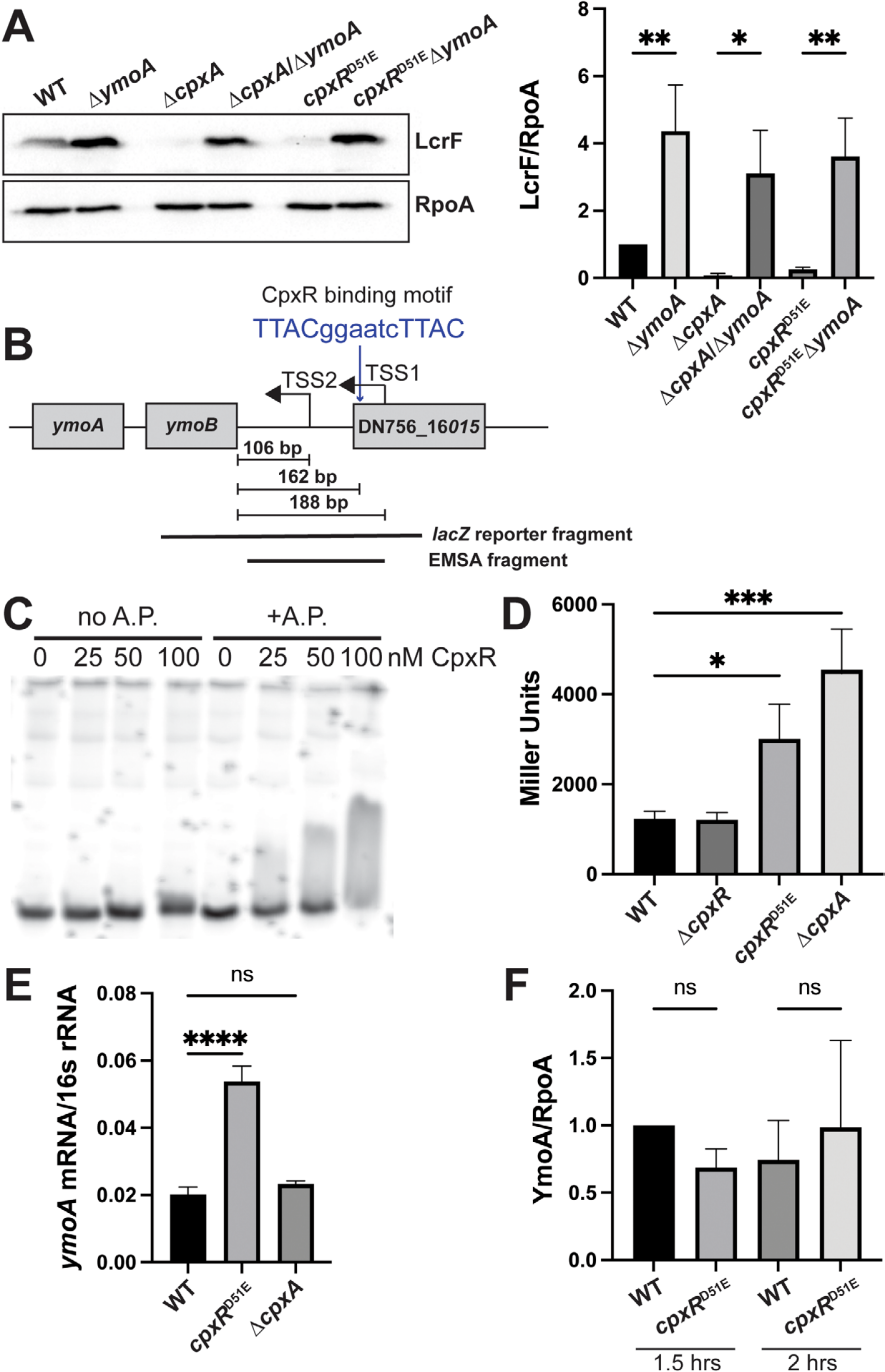
CpxR binds to the *ymoA* promoter and YmoA is required for CpxR‐dependent inhibition of T3SS expression, but CpxR activation does not alter YmoA protein levels. (A) *Y. pseudotuberculosis* strains were grown in T3SS‐inducing conditions, normalized for cell density, and whole cell lysates collected for western blotting using LcrF‐ and RpoA‐specific antibodies. Densitometry was used to measure the relative amount of LcrF relative to the RpoA control. Average ± StDev of three independent experiments is shown. (B) Bioinformatic analysis revealed a putative CpxR binding site in the *ymoB‐ymoA* promoter 56 bp upstream of TSS2. (C) EMSA performed with purified CpxR incubated with and without acetyl phosphate (A.P.) as the phosphodonor and a PCR‐amplified 200 bp fragment ranging from −94 to +106 relative to TSS2 of *ymoB*. (D) *Y. pseudotuberculosis* strains containing plasmid pFU99 encoding a *lacZ* gene driven by the *ymoBA* promoter (−194 to +136 relative to *ymoB* TSS2) were grown in T3SS‐inducing conditions, lysed, and assessed for β‐galactosidase activity by addition of ONPG. Average ± StDev of three independent experiments is shown. (E) *Y. pseudotuberculosis* strains were grown in T3SS‐inducing conditions and mRNA was isolated for qPCR analysis. *ymoA* mRNA levels were normalized to 16s rRNA. Average ± StDev of three independent experiments is shown. (F) Wildtype and *cpxR* ^D51E^ *Y. pseudotuberculosis* were grown in low calcium LB at 26°C for 1.5 h before being shifted to 37°C to induce the T3SS. Samples were taken at 1.5 and 2 h post‐shift to 37°C. YmoA protein levels were assayed via western blot. Densitometry was used to measure the relative amount of YmoA relative to the RpoA control. Average ± StDev of three independent experiments is shown. *p < 0.05, **p < 0.01, ***p < 0.001, ****p < 0.0001 (one‐way ANOVA with Tukey comparison). A representative image is shown in Figure S3.

To address how CpxR regulates T3SS expression, we investigated the potential involvement of CpxR in regulation of YmoA, a co‐repressor of the H‐NS architectural binding protein (Balderas et al. 2022; Böhme et al. 2012). Interestingly, deletion of *ymoA* in both the Δ*cpxA* and *cpxR* ^D51E^ genetic backgrounds led to LcrF derepression (Figure 5A), suggesting that CpxAR‐mediated repression of the T3SS is dependent on YmoA, perhaps by controlling *ymoA* expression. The *ymoB‐ymoA* operon was shown to have two TSSs, one 188 bp upstream of the *ymoB* start codon (TSS1) and another 106 bp upstream of the *ymoB* start codon (TSS2) (Nuss et al. 2015) (Figure 5B). We bioinformatically identified one highly conserved CpxR binding motif 162 bp upstream of the *ymoB* start codon (Figure 5B, *p* = 2.08 × 10^−5^). Binding of purified CpxR~*P* to a 200 bp *ymoBA* promoter fragment containing the putative CpxR binding motif was observed via EMSA (Figure 5C) under the same conditions we observed binding to the *cpxR* promoter (Figure 4C). To determine whether CpxR regulates *ymoBA* promoter activity, promoter activity assays were performed. A construct containing the putative CpxR binding site upstream of TSS2 increased activity in the ∆*cpxA* and *cpxR* ^D51E^ mutants relative to wildtype (Figure 5D). This increase in promoter activity was consistent with an increase in *ymoA* mRNA levels in the *cpxR* ^D51E^ mutant compared to wildtype (Figure 5E), although this was not observed in the Δ*cpxA* mutant, possibly due to the growth defect of the Δ*cpxA* strain. While these data are consistent with a role for YmoA in CpxR‐mediated T3SS repression, YmoA protein levels were similar between the wildtype and *cpxR* ^D51E^ strains (Figure 5F, Figure S3). Taken together, these data suggest that while CpxR~*P* may bind to the *ymoBA* promoter, CpxR~P does not repress the *yscW‐lcrF* promoter by increasing YmoA protein levels.

### 2.5 RNA‐Seq Analysis of Strains With Constitutively Activated CpxR Reveals That OmpR Regulates the *Y. pseudotuberculosis* T3SS

**FIGURE 6:**
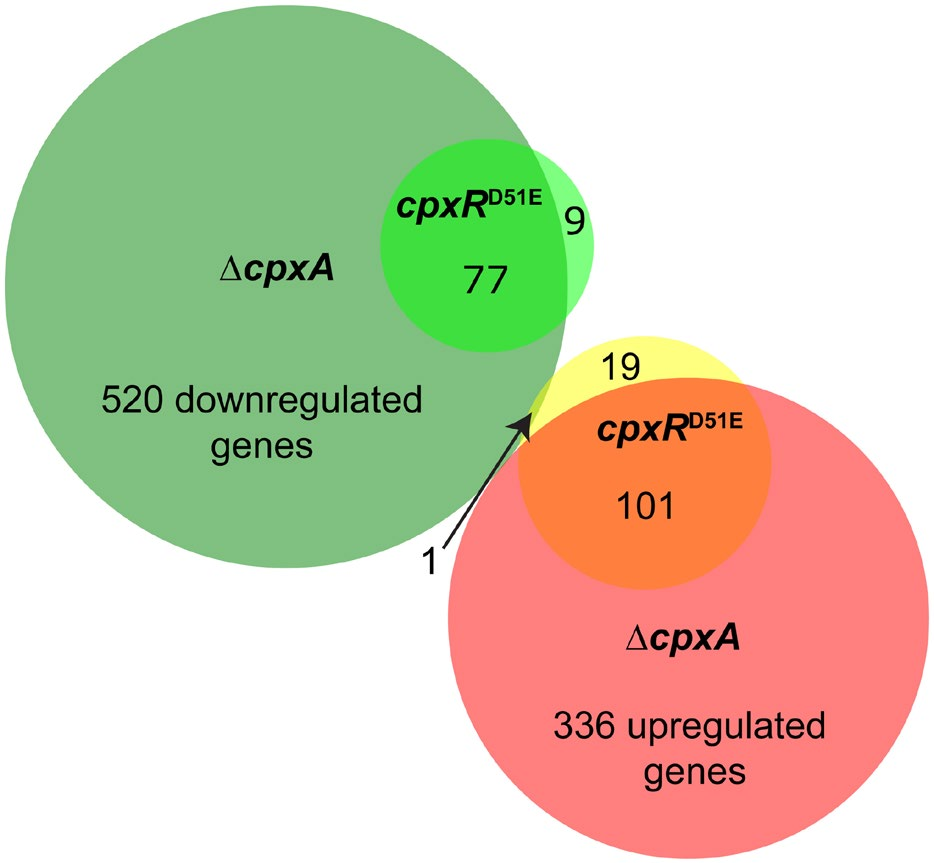
Summary of differential gene expression in Δ*cpxA* and *cpxR* ^D51E^ mutants relative to wildtype *Y. pseudotuberculosis* under T3SS‐inducing conditions. *Y. pseudotuberculosis* wildtype, ∆*cpxA*, and *cpxR* ^D51E^ strains were grown under T3SS‐inducing conditions and differential gene expression was determined by RNA‐Seq analysis. The Venn diagram was created using DeepVenn (Hulsen 2022) and shows the number of genes differentially expressed between the ∆*cpxA* or *cpxR* ^D51E^ strains compared to wildtype.

**FIGURE 7:**
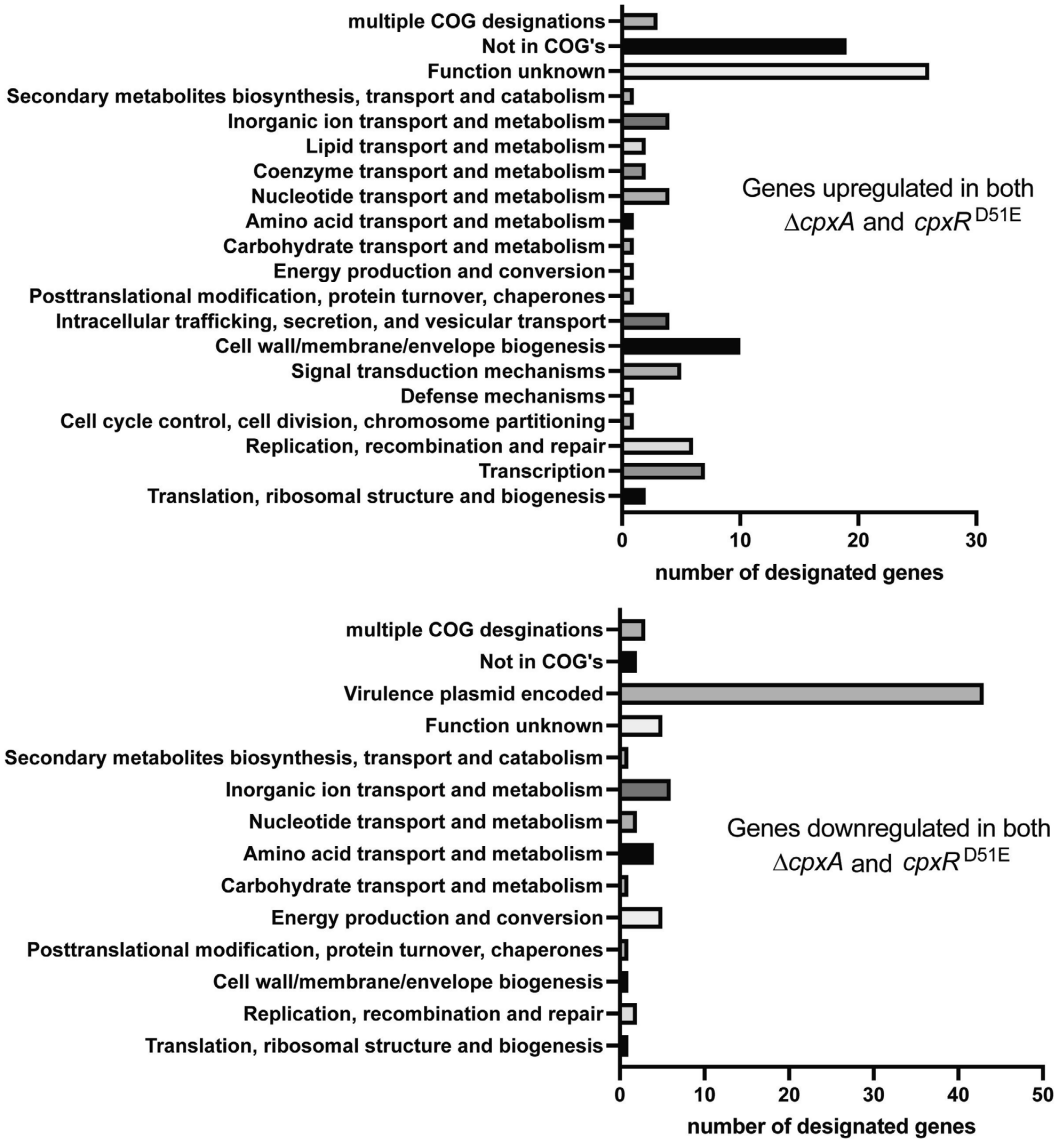
Categories of Orthologous Groups (COG) analysis of differentially expressed genes in both the Δ*cpxA* and *cpxR* ^D51E^ mutants compared to wildtype. COG analysis was carried out on the 101 upregulated (A) and 77 downregulated (B) genes in both the ∆*cpxA* and *cpxR* ^D51E^ strains relative to wildtype.

While CpxAR is among the most studied TCS in Gram‐negative bacteria (Choudhary et al. 2020; Price and Raivio 2009; Zhao et al. 2022), extensive characterization of its regulon has not been performed in *Yersinia* or with CpxR‐activated mutants, nor has the regulon been explored under conditions that mimic pathogen‐host cell contact. We carried out RNA‐seq analysis to identify differentially expressed genes in wildtype, Δ*cpxA*, and *cpxR* ^D51E^ *Y. pseudotuberculosis* strains grown under T3SS‐inducing conditions. The *ΔcpxA* and *cpxR* ^D51E^ strains displayed 1035 and 207 differentially expressed genes compared to wildtype, respectively (Figure 6, Dataset S1, GEO series accession number GSE313348). Because both the *ΔcpxA* and *cpxR* ^D51E^ strains exhibited decreased T3SS expression, we focused on genes that were differentially expressed in both mutants. Of these, 101 genes were upregulated and 77 genes were downregulated relative to wildtype. These genes were sorted into categories of orthologous groups (COGs) as previously described (Balderas et al. 2021) (Figure 7A,B). As expected, genes involved in cell wall or membrane biogenesis and signal transduction were upregulated in both the ∆*cpxA* and *cpxR* ^D51E^ mutants compared to wildtype. Since our results suggested that the defect in T3SS expression exhibited by the ∆*cpxA* and *cpxA* ^D51E^ mutants could be rescued by overexpressing LcrF and that CpxR~*P* led to decreased *yscW‐lcrF* promoter activity, we focused our attention on predicted transcriptional regulators that were differentially expressed in the ∆*cpxA* and *cpxA* ^D51E^ mutants compared to wildtype. Of these, seven genes with appreciable TMM values were identified as likely to be involved in transcriptional regulation (Table 1). One gene (*pspA*) was downregulated in each mutant relative to wildtype *Y. pseudotuberculosis* , while the others were upregulated.

**TABLE 1:** Select genes found to be differentially expressed between both the ∆*cpxA* and *cpxR* ^D51E^ mutants compared to wildtype.

Locus tag	Gene name	Description
DN756_09320	*pspA*	Phage shock protein A, PspF‐binding protein
DN756_02835	*mzrA*	Modulates the activity of the EnvZ OmpR two‐component regulatory system
DN756_14435	*zraR*	Sigma‐54 interaction domain
DN756_16420	*crl*	Binds to the sigma‐S subunit of RNA polymerase, activating expression of sigma‐S‐regulated genes.
DN756_07600	*lrhA*	LysR substrate binding domain
DN756_10150	DN756_10150	Transcription factor zinc‐finger
DN756_06880	DN756_06880	Transcriptional regulatory protein, C terminal

**FIGURE 8:**
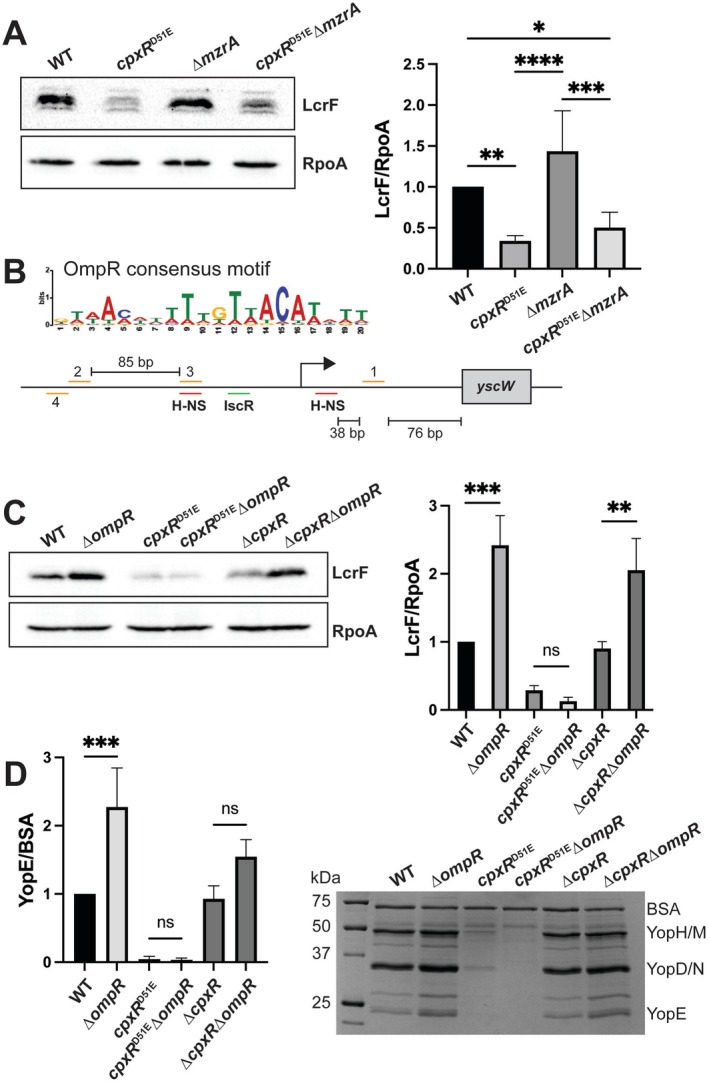
OmpR is a negative regulator of LcrF and T3SS expression in *Y. pseudotuberculosis* . (A) *mzrA*, a CpxR‐responsive gene, was deleted in *Y. pseudotuberculosis* wildtype and *cpxR* ^D51E^ strain backgrounds and LcrF levels were assessed via western blot. Average ± StDev of five independent experiments is shown. (B) A training set containing experimentally confirmed OmpR binding sites in *E. coli* , *Salmonella*, and *Yersinia pestis* was used to generate an OmpR consensus binding motif using MEME Suite. A bioinformatic search using this consensus motif identified four possible OmpR binding sites in the *yscW‐lcrF* promoter (site 1 *p* = 0.000118; site 2 *p* = 0.000176; site 3 *p* = 0.00027; site 4 *p* = 0.000639). One higher *p*‐value site (*p* = 0.00962) was excluded due to substantial overlap with site 1. (C, D) The *ompR* gene was deleted in wildtype, *cpxR* ^D51E^, and Δ*cpxR* strain backgrounds, and LcrF protein levels (C) and T3SS activity (D) assessed. Average ± StDev of three independent experiments is shown. *p < 0.05, **p < 0.01, ***p < 0.001, ****p < 0.0001 (one‐way ANOVA with Tukey comparison).

Deletion mutations of each selected gene were constructed in wildtype and *cpxR* ^D51E^ strain backgrounds. Western blots were performed on each mutant to compare LcrF levels relative to parental strains (Figure S4). Of the seven mutants, only Δ*mzrA* trended toward recovering LcrF levels in the *cpxR* ^D51E^ background, although this was not statistically significant (Figure 8A). A putative CpxR‐binding site was identified approximately 200 bp upstream of the nearest TSS relative to *mzrA* (*p* = 9.67 × 10^−5^) (Nuss et al. 2015). MzrA does not bind DNA itself but instead has been shown in *E. coli* to modulate the activity of the osmolarity stress response protein OmpR, via an interaction with the EnvZ histidine kinase, which results in higher levels of OmpR~*P* (Gerken et al. 2009). We bioinformatically identified four potential OmpR binding sites in the *yscW‐lcrF* regulatory region (Figure 8B). To determine if OmpR regulates LcrF expression, Δ*ompR* mutants were constructed in WT, *cpxR* ^D51E^, and Δ*cpxR* backgrounds. Both LcrF protein levels (Figure 8C) and YopE secretion (Figure 8D) were significantly elevated in Δ*ompR* compared to wildtype, suggesting that OmpR acts as a negative regulator of LcrF expression. While deletion of *ompR* in the *cpxR* ^*D51E*^ background was unable to rescue LcrF expression, the growth defect of the ∆*ompR*/*cpxR* ^D51E^ strain complicates interpretation of this result (Figure S5). Indeed, the complete lack of T3SS activity in this strain suggests additional pleiotropic effects of this double mutation. Taken together, our data suggest that CpxR may repress LcrF indirectly through multiple redundant pathways, and that OmpR may be a novel repressor of LcrF.

## 3 Discussion

While the T3SS is required for *Yersinia* virulence, its expression is not essential for colonization of all host tissues (Crimmins et al. 2012; Hooker‐Romero et al. 2019) and may even be detrimental for *Yersinia* colonization of certain tissues (Ohanyan et al. 2025). This requires *Yersinia* to incorporate environmental signals to selectively express the T3SS, for example, by regulating transcription of *lcrF* by the iron and oxygen sensor IscR in response to aerobic low iron conditions (Balderas et al. 2022; Hooker‐Romero et al. 2019; Miller et al. 2014). Our study suggests that *Yersinia* sensing of other environmental cues through CpxR and OmpR may also modulate T3SS expression.

Our data suggest that while activation of CpxR leads to decreased expression of the *Yersinia* T3SS master regulator LcrF, we could not find evidence that CpxR binds strongly to the *yscW‐lcrF* regulatory regions. In contrast, previously published work suggested that at ~10‐fold higher concentrations, CpxR~*P* binds to the intergenic region of *yscW‐lcrF* (Liu et al. 2012); no binding to this intergenic region or the well‐defined promoter region upstream of the *yscW‐lcrF* operon (Nuss et al. 2015) was observed in this study at CpxR~*P* levels sufficient to bind a known binding site. It is possible that the extent of CpxR phosphorylation and/or our specific reaction conditions may account for the discrepancy between our results and the previous work. However, if very high levels of CpxR~*P* are needed to bind to the *yscW‐lcrF* intergenic region, it is unlikely to impact *lcrF* expression since *lcrF* transcription is driven from the promoter upstream of *yscW*. Investigation of promoter elements of additional genes known to be involved in LcrF regulation revealed that while CpxR bound to the promoter of the *yscW‐lcrF* co‐repressor YmoA, constitutive CpxR activation did not affect YmoA protein levels. We conclude that activated CpxR likely indirectly represses *yscW‐lcrF* transcription, and therefore T3SS expression, through multiple redundant pathways. Our RNA‐seq and bioinformatic analysis of *Yersinia* strains with constitutive CpxR activation led us to identify OmpR as a novel regulator of LcrF.

Previously published machine learning‐based data in *E. coli* suggests that CpxR is a regulator of the gene *tomB* (Choudhary et al. 2020). In *Yersinia*, *tomB/ymoB* is the first gene in a polycistronic operon with the gene encoding the *lcrF* negative regulator YmoA. In our investigation of *Yersinia* CpxR regulation of *ymoA*, we found that phosphorylated CpxR binds to the *ymoB‐ymoA* promoter and activation of CpxR increased *ymoB‐ymoA* promoter activity. However, we did not detect differential expression of *ymoA* in either the Δ*cpxA* or *cpxR* ^D51E^ mutants compared to wildtype by RNA‐Seq analysis, and YmoA protein levels were unchanged between wildtype and the *cpxR* ^D51E^ mutant. This was surprising given our qPCR data showing elevated *ymoA* mRNA levels in *cpxR* ^D51E^ after 1.5 h at 37°C, so YmoA protein levels were also assayed at 2 h after the shift to 37°C to account for any possible delays between the increase in transcription and the reflection of this upregulation in YmoA steady‐state protein levels. Even at this later timepoint no increase in YmoA protein levels was observed. Therefore, we conclude that the ability of activated CpxR to repress LcrF expression is likely independent of *ymoB‐ymoA* promoter regulation. As YmoA acts with H‐NS as a global transcriptional regulator in *Yersinia*, it is possible that CpxR~*P*‐driven *ymoB‐ymoA* transcription may be relevant under environmental conditions unrelated to T3SS expression.

Regulation of YmoA is complex and in need of further research. While incubation of *Yersinia* at 37°C leads to an increased rate of degradation of YmoA protein (Jackson et al. 2004), it is evident that some amount of YmoA still acts alongside H‐NS to repress *yscW‐lcrF* transcription at host body temperature under T3SS‐inducing conditions (Balderas et al. 2022). It is possible that *ymoA* mRNA may be post‐transcriptionally regulated under T3SS‐inducing conditions, and that this buffers YmoA protein levels from any CpxR‐driven changes in *ymoA* mRNA levels.

Our RNA‐Seq analysis of *Y. pseudotuberculosis* strains with constitutively activated CpxR identified a number of CpxR‐regulated genes, including those implicated in transcriptional regulation. One identified gene encodes the protein MzrA, a modulator of the osmolarity response system EnvZ‐OmpR that is thought to promote increased levels of the active phosphorylated form of the response regulator OmpR through interaction with the EnvZ histidine kinase (Gerken et al. 2009). The EnvZ‐OmpR TCS responds primarily to environmental osmolarity and pH stress (Kenney and Anand 2020). Low osmolarity or neutral pH conditions lead to a low level of EnvZ activity and a low rate of OmpR phosphorylation. Low levels of active OmpR lead to preferential binding of sites that more closely match the consensus sequence and expression of genes required for these conditions, such as the F1‐F2‐F3 sites of the low osmolarity porin gene *ompF*. Under conditions of high osmolarity or acidic pH, EnvZ activity increases and results in a larger population of phosphorylated OmpR in the cytoplasm, leading to occupation of additional OmpR‐binding sites in the genome with less similarity to the consensus. This results in repression of *ompF* via occupation of the F4 binding site in the *ompF* promoter, as well as activation of the high osmolarity porin gene *ompC* (Harlocker et al. 1995; Kenney and Anand 2020; Maeda and Mizuno 1990; Ostrow et al. 1986; Rampersaud et al. 1994; Russo and Silhavy 1991; Slauch et al. 1988; Slauch and Silhavy 1989; Taylor et al. 1983). Under low osmolarity conditions that favor unphosphorylated OmpR, OmpF levels should be high and OmpC levels low. Conversely, under high osmolarity conditions, OmpR phosphorylation should increase and OmpC should be the predominant porin expressed. Under the T3SS‐inducing conditions used in our study, *ompC* TMM values were approximately 13‐fold higher than *ompF* TMM values in wildtype *Y. pseudotuberculosis* (Figure S6), suggesting that OmpR~*P* levels are likely elevated. MzrA has been previously identified as a member of the CpxR regulon and represents a link between CpxR and OmpR activity (Gerken et al. 2009). This suggests that an upregulation of *mzrA* in strains with constitutive CpxR activity should lead to increased OmpR~P levels and higher *ompC*, but we did not observe this (Figure S6). It is possible that OmpR‐mediated regulation of *ompF* and *ompC* does not follow the same patterns in *Y. pseudotuberculosis* as in *E. coli* . Indeed, it has been suggested that the closely related organism, *Yersinia pestis* , lacks the *ompF* promoter site F4, which mediates OmpR‐dependent repression of *ompF* in conditions of high osmolarity (Gao et al. 2011). It is likewise possible that there are non‐OmpR regulators of the *ompF* and *ompC* promoters that may alter overall expression patterns in these conditions.

While deletion of *ompR* in the *cpxR* ^D51E^ background did not restore LcrF levels, interpretation of these data was complicated by the growth defect of this strain as well as its complete lack of T3SS activity. However, deletion of *ompR* in the wildtype and Δ*cpxR* mutant backgrounds led to an increase in LcrF levels, suggesting that OmpR directly or indirectly represses LcrF and that this does not require CpxR. While OmpR has been implicated as a regulator of the Ysc T3SS in *Yersinia enterocolitica* (Nieckarz et al. 2022), OmpR‐mediated effects on secretion were independent of the T3SS master regulator in that species, VirF. This study therefore demonstrates an as‐yet unappreciated function of OmpR as a regulator of LcrF and the Ysc T3SS. As with other environmentally‐sensitive regulators of LcrF including YmoA/H‐NS, IscR, and CpxR, the addition of OmpR as a repressor of the T3SS provides interesting implications for the progression of *Yersinia* virulence. Throughout infection, *Yersinia* is exposed to a variety of environments, starting with the gastrointestinal tract before dissemination into deeper tissues such as the spleen and liver (Hooker‐Romero et al. 2019). As a pH and osmolarity sensor, OmpR may contribute to T3SS modulation in host tissues along with IscR, which senses changes in iron availability and oxygen tension to directly control the *yscW‐lcrF* promoter (Hooker‐Romero et al. 2019). For example, transit through the intestinal tract is expected to expose bacteria to low pH and high osmolarity stress along with hypoxia and iron availability. *Yersinia* does not require its T3SS for colonization of the intestinal tract (Hooker‐Romero et al. 2019), and future studies will address whether IscR and OmpR both contribute to repression of the *Yersinia* T3SS in the intestine. Conversely, OmpR and IscR sensing of the environment encountered by *Yersinia* during extraintestinal infection (i.e., ‐neutral pH, lower osmotic stress sensed by OmpR; low iron availability, oxygen sensed by IscR) may promote *lcrF* derepression in tissue microenvironments where *Yersinia* requires the T3SS to subvert phagocyte effector functions.

The RNA‐seq analysis presented here has additional implications for the study of the CpxAR TCS. Previously published studies have commonly used *cpxA* null mutants as a model for Cpx system activation in different organisms (Carlsson et al. 2007a; Danese et al. 1995; Liu et al. 2012; Spinola et al. 2010; Subramaniam et al. 2019). While it has been demonstrated that this strain contains an increased pool of phosphorylated CpxR (Liu et al. 2011), our study indicates that there may be pleiotropic effects caused by this mutation in *Y. pseudotuberculosis* . The five‐fold increase in the number of differentially expressed genes in the Δ*cpxA* mutant compared to the *cpxR* ^*D51E*^ phosphomimic mutant relative to wildtype, as well as the more severe growth defect in the Δ*cpxA* mutant, suggests that the *Y. pseudotuberculosis* Δ*cpxA* mutant may not represent a straightforward model of CpxR activation. In contrast, the *cpxR* ^*D51E*^ mutant has a much less severe growth defect yet still exhibits substantial CpxR activity. There are several possible explanations for the differences between these two strains. A previous study suggested that the Δ*cpxA* mutant not only has a larger overall pool of CpxR than the WT strain, but that this pool is a mix of unphosphorylated CpxR and CpxR~*P* (Liu et al. 2012). Unphosphorylated CpxR has not been definitively shown to act in a regulatory manner, but some response regulators have regulatory roles outside of their phosphorylated state (Desai and Kenney 2017; Kenney and Anand 2020). In comparison, the *cpxR* ^D51E^ mutant only expresses CpxR in the phosphomimic state. In addition, it has also been suggested that TCSs can engage in cross‐talk, with the histidine kinase engaging in kinase or phosphatase activity with proteins that are not its cognate response regulator (Kenney and Anand 2020; Siryaporn and Goulian 2008). Deletion of the *cpxR* gene in the ∆*cpxA* strain background rescues its growth defect, suggesting that the impact of the ∆*cpxA* mutation on *Yersinia* growth is dependent on CpxR. However, the high degree of homology between many different response regulators, as well as the very high levels of CpxR~*P* present in the Δ*cpxA* mutant, may result in cross‐talk of CpxR~P with other histidine kinases. This could hinder the normal activity of other TCSs, impacting bacterial growth and/or gene expression. While the mechanisms behind the discrepancies in growth and differentially expressed genes between these two strains remain unclear, the evident high degree of stress in the Δ*cpxA* mutant presents an opportunity to study general disruption of bacterial membrane homeostasis.

These data also open questions related to the synergy of different membrane stress responses in the regulation of the T3SS. For instance, while CpxR primarily responds to inner membrane and periplasmic stress signals, both the Cpx and Rcs TCSs can respond to similar stressors in *Yersinia* spp. and *Salmonella*. For example, exposure to cationic antimicrobial peptides such as polymyxin B is associated with both Cpx and Rcs responses in *Yersinia* spp. (Meng et al. 2019; Zhou et al. 2006) and *Salmonella* (Erickson and Detweiler 2006; Subramaniam et al. 2019). Oxidative stress has likewise been associated with both Cpx and Rcs responses in *Salmonella* (Farizano et al. 2014; López et al. 2018). Interestingly, the outer membrane stress response protein RcsB has been implicated as an activator of T3SS expression in *Yersinia* (Li et al. 2015), although the physiological relevance of this interaction is unclear. As the same environmental signals may be activating these responses, it is unclear why RcsB would act as a positive regulator of LcrF while CpxR acts as an indirect negative regulator. Furthermore, CpxR has been implicated as a negative regulator of RcsB (Fei et al. 2021), although in our RNA‐Seq analysis *rscB* was not differentially expressed between the ∆*cpxA* or *cpxR* ^D51E^ mutants and wildtype (Figure S7).

Overexpression of the lipoprotein NlpE, which has been shown to activate the CpxAR two‐component system and increase overall CpxR levels as well as levels of phosphorylated CpxR (Danese et al. 1995; Delhaye et al. 2019; Liu et al. 2012; Snyder et al. 1995), reduces LcrF levels and T3SS activity in *Yersinia*. While the effect of NlpE overexpression on LcrF levels was CpxR‐dependent, the effect on T3SS activity was not. These data suggest that activation of CpxR by lipoprotein mislocalization represses LcrF expression. However, since overexpression of lipoproteins disrupts lipoprotein localization to the outer leaflet of the outer membrane (Delhaye et al. 2019; May et al. 2019) and the lipoprotein YscJ is critical for assembly of the *Yersinia* Ysc T3SS (Deng et al. 2017), it is possible that NlpE overexpression also disrupts proper T3SS assembly and/or activity independently of CpxR. The role that CpxR regulation of the *Yersinia* T3SS plays in *Yersinia* virulence remains unclear. While serotonin, which is present at high levels in the intestine, has been shown to decrease CpxA phosphorylation and CpxR activation in enterohemorrhagic *E. coli* and *Citrobacter rodentium* (Kumar et al. 2020), serotonin did not relieve repression of T3SS activity in response to NlpE activation in *Y. pseudotuberculosis* (Figure S8). Another CpxR activation signal with possible relevance to infection is copper, as elevated copper levels block normal lipoprotein trafficking via the Lol system (May et al. 2019). Copper is imported into the macrophage phagosomes as an antimicrobial mechanism (White et al. 2009). However, the Ysc T3SS blocks uptake of *Yersinia* into macrophages, and *Yersinia* is thought to reside largely in the extracellular compartment during infection (Pha and Navarro 2016). While some strains of *Y. pseudotuberculosis* can replicate inside macrophages (Pujol and Bliska 2003), the significance of intracellular *Yersinia* remains unclear. It is possible that in serving as a sensor of perturbations to periplasmic homeostasis such as lipoprotein overproduction and/or mislocalization, the CpxAR system tunes T3SS expression levels to optimize levels of T3SS structural proteins in the periplasm. Our data suggest that the CpxAR TCS system may accomplish this through multiple parallel pathways that ultimately act to downregulate *yscW‐lcrF* promoter activity. One of those pathways may be mediated by OmpR, whose activity is regulated by osmolarity and low pH (Kenney and Anand 2020). As bacteria encounter significant acid stress and high osmolarity conditions in the digestive tract (Sleator et al. 2007), where T3SS expression is not required for colonization and may be detrimental (Hooker‐Romero et al. 2019; Ohanyan et al. 2025), CpxR and OmpR may enable *Yersinia* to sense its biogeography to tune T3SS expression.

## 4 Materials and Methods

### 4.1 Bacterial Strains, Plasmids, and Growth Conditions

All bacterial strains used in this study are listed in Table S1. Plasmids used in this study are listed in Table S2. *Y. pseudotuberculosis* strains were grown in LB (Luria broth) media at 26°C and shaking at 250 rpm unless otherwise indicated. Low calcium LB media was made by supplementing LB with sodium oxalate to a final concentration of 20 mM to chelate calcium ions and 20 mM MgCl_2_ to ensure sufficient levels of magnesium in the media. For experiments carried out in T3SS‐inducing conditions, *Y. pseudotuberculosis* grown in LB overnight was subcultured to an optical density (OD_600_) of 0.2 in fresh low calcium LB. Cultures were then grown at 26°C shaking for 1.5 h and then moved to 37°C shaking for 1.5 h to induce type III secretion (Auerbuch et al. 2009). Western blots probing YmoA levels were additionally sampled at 2 h after shifting to 37°C. For overexpression constructs, arabinose or IPTG was added to cultures during the subculturing step prior to incubation at 26°C from 20% arabinose or 10 mM IPTG stocks for LcrF and NlpE overexpression, respectively, and vortexed briefly.

### 4.2 Construction of *Y. pseudotuberculosis* Mutant Strains

Primers used in the construction of mutant strains are listed in Table S3. *Y. pseudotuberculosis* mutants were generated by allelic exchange using pSR47s suicide plasmids containing ~1000–2000 bp of homology around allelic exchange target sites. Deletion strains were made by amplifying and cloning ~1000 bp fragments up and downstream of the gene of interest and assembling into the pSR47s backbone using the NEBuilder HiFi DNA Assembly kit (New England Biolabs Inc). The genes *mzrA* and *zraR* overlap with other open reading frames in *Yersinia*. Those overlapping ORFs were preserved in the *mzrA* and *zraR* deletion mutants. Point mutations were made by cloning a ~1000 bp fragment centered on the site of interest into a pSR47s backbone and subsequently performing Q5 mutagenesis to generate the desired mutation.

Suicide plasmids were transformed into *E. coli* S17‐1 λpir competent cells and later conjugated into *Y. pseudotuberculosis* . The resulting kanamycin‐ and irgasan‐resistant clones were then grown in liquid culture in the absence of antibiotic and plated on sucrose‐containing media to select for clones lacking the *sacB* gene. Kanamycin‐sensitive, irgasan‐resistant, and Congo red‐positive or negative (as applicable) colonies were screened by PCR and sequence confirmed. Congo red is a T3SS indicator dye that is selectively taken up by *Yersinia* with active type III secretion (Riley and Toma 1989).

### 4.3 Growth Curves

*Y. pseudotuberculosis* strains were grown overnight at 26°C in LB. For growth curves in standard *Y. pseudotuberculosis* growth conditions, strains were subcultured to an OD_600_ of 0.2 in fresh LB and grown shaking at 26°C for 8 h. Every hour, the OD_600_ was measured using a WPA biowave C08000 Cell Density Meter to track bacterial growth. For growth in T3SS‐inducing conditions, strains were subcultured to an OD_600_ of 0.2 in low calcium LB and allowed to grow at 26°C shaking for 1.5 h before being shifted to 37°C to induce type III secretion. Cultures were shaken at 37°C for 4–7 h and OD_600_ measured every hr.

### 4.4 β‐Galactosidase Promoter Activity Assays

Promoter activity reporter plasmids were constructed in pFU99a backbones using primers listed in Table S3. Promoter activity assays were performed in T3SS‐inducing conditions (low calcium LB, 1.5 h at 26°C, followed by 1.5 h at 37°C, as previously described (Balderas et al. 2022)). Briefly, samples were incubated on ice to halt protein expression before being pelleted and resuspended in Z buffer and permeabilized using chloroform and 0.1% SDS. Samples were then incubated with 4 mg mL^−1^ ONPG. β‐galactosidase activity was halted by addition of 1 M sodium bicarbonate and promoter activity was assessed by changes in OD_420_ normalized to OD_550_ and OD_600_ using a Thermo‐Scientific Genesys 150 spectrophotometer. β‐galactosidase activity is reported as Miller units, defined as [1000 (OD_420_ − 1.75 × OD_550_)]/(ONPG incubation time in minutes × volume × OD_600_).

### 4.5 RNA Isolation and Quantitative PCR (qPCR)

*Y. pseudotuberculosis* was grown in T3SS‐inducing conditions. Cultures were then pelleted and stabilized with Qiagen RNA Protect Bacterial Reagent (76506). Total RNA was isolated using the Qiagen RNeasy Mini kit (74106) and DNA was removed from samples using the Thermo Fisher TURBO DNA‐free Kit (AM1907). cDNA was generated using Invitrogen M‐MLV Reverse Transcriptase (28025) and diluted to optimal concentrations for each gene probed. RT‐qPCR was performed using 7.5 μL diluted cDNA and 7.5 μL Power SYBR Green PCR Master Mix (4367659) containing 1.67 mM forward and reverse primers (Table S3) and run using a Biorad CFX Connect Real‐Time System. qPCR data were analyzed using the standard curve method with 16 s rRNA as the normalizing housekeeping gene.

### 4.6 T3SS Secretion Assays

Visualization of secreted T3SS proteins was performed as previously described (Kwuan et al. 2013). *Y. pseudotuberculosis* was grown in low calcium LB media for 1.5 h at 26°C followed by 1.5 h at 37°C. Cultures were normalized by OD_600_ and supplemented with BSA (as a protein precipitation control) before being pelleted at 21,130 rcf for 15 min at room temperature. The supernatants were transferred to fresh tubes and the secreted proteins were precipitated using trichloroacetic acid (TCA), which was added at a final concentration of 10%. Samples were incubated on ice overnight. Precipitated supernatants were then pelleted by centrifugation at 21,130 rcf for 15 min at 4°C and washed twice with cold acetone. Proteins were then resuspended in Final Sample Buffer (FSB) with 200 mM dithiothreitol (DTT). Samples were boiled for 15 min at 95°C prior to running on a 12.5% SDS‐PAGE gel, which was then stained with Coomassie Brilliant Blue to visualize proteins. The ~23 kDa T3SS secreted protein YopE was quantified as a proxy for type III secretion efficacy and normalized to the BSA control as previously described (Balderas et al. 2022, 2021; Lam et al. 2021; Ohanyan et al. 2024). Visualization and quantification were performed using BioRad Image Lab software.

### 4.7 Western Blot Analysis

Bacteria were grown in T3SS‐inducing conditions and pelleted by centrifugation. Pellets were resuspended in FSB + DTT and boiled for 10 min at 95°C prior to running on either a 16.5% Tris‐tricine gel (YmoA visualization) or a 12.5% SDS‐PAGE gel (all other proteins). Proteins were then transferred onto nitrocellulose membranes and blocked with Tris‐buffered saline with Tween 20 (TBST) solution and 5% skim milk overnight at 4°C. Membranes were likewise incubated with primary antibody in 5% skim milk TBST solution overnight at 4°C. Membranes were then washed in fresh TBST three times before incubating with secondary antibody for 1 h, washed, and incubated for 1 min in western blotting Luminol Reagent. Bands were visualized and quantified using BioRad Image Lab. Primary antibodies include rabbit anti‐RpoA (gift from Melanie Marketon), rabbit anti‐LcrF (gift from Gregory Plano), rabbit anti‐CpxR (gift from Matthew Francis), and rabbit anti‐YmoA (gift from Gregory Plano). Mouse‐anti‐rabbit antibody conjugated with horseradish peroxidase was obtained from Santa Cruz Biotech (sc‐2357).

### 4.8 RNA‐Seq Analysis

Total mRNA was isolated and DNase treated in the same manner as described above. RNA quality was assessed via Bioanalyzer. RNA‐sequencing and analysis was performed by SeqCenter in Pittsburgh, PA. Briefly, DNase (Invitrogen) treatment was repeated and subsequent library preparation was done using the Illumina Stranded Total RNA Prep Ligation with Ribo‐Zero Plus kit and 10 bp unique dual indices. Sequencing was then performed on a NovaSeq X Plus to produce paired end 150 bp reads. bcl‐convert (v4.2.4) was used for demultiplexing, quality control and adapter trimming. HISAT2 was used for read mapping. Read counts were normalized using the TMM method and converted into counts per million, which was used for differential expression analysis using glmQLFTest in edgeR. Calls for differentially expressed genes were restricted to those with |logFC| > 1 and *p* < 0.05. RNA sequences were mapped to *Y. pseudotuberculosis* strain IP2666pIB1 GenBank annotations under accession numbers CP032566.1 (chromosome) and CP032567.1 (pYV virulence plasmid).

### 4.9 Bioinformatic Analysis of Transcription Factor Binding Sites

We generated a consensus binding motif for OmpR using MEME (Bailey and Elkan 1994) and a list of *E. coli* OmpR binding sites from RegulonDB, confirmed binding sites from *Yersinia pestis* (Gao et al. 2011), and ChIP‐Seq data from *E. coli* and *Salmonella* (Quinn et al. 2014). The motif generated resembled that of the consensus motif elucidated by Maeda et al. (1991), Nieckarz et al. (2016). This motif was used to scan 575 bp upstream of the *yscW* start codon using MEME Suite FIMO (Bailey and Elkan 1994). For CpxR, training sets were obtained from RegulonDB and binding motifs were generated using MEME as previously described (Balderas et al. 2022). MEME Suite FIMO was then used to search for motifs in the *ymoB‐ymoA* promoter region or *yscW‐lcrF* promoter and intergenic regions as described.

### 4.10 CxpR‐6xHis Purification

*E. coli* containing pET28b‐*cpxR*‐6xHis was grown in 1 L of LB broth shaking 250 rpm at 37°C to an OD_600_ of ~0.6 before chilling and addition of IPTG to a final concentration of 0.2 mM and overnight induction of protein expression at 18°C. Cells were then pelleted and resuspended in Lysis Buffer (10 mM imidazole, 200 mM NaCl, 50 mM Tris pH 8.0) with 1× PMSF complete protease inhibitor. Cells were lysed via sonication at 20 s intervals until smooth. Lysate was then centrifuged for 25 min at 42,000 rcf after which the supernatant was added to a gravity column containing Ni‐agarose His‐tag affinity beads. The column was subsequently washed with Wash Buffer (200 mM NaCl, 50 mM Tris pH 8.0) with increasing concentrations of imidazole (10, 30, 50 mM). The protein was eluted with Elution Buffer (250 mM imidazole, 200 mM NaCl, 50 mM Tris pH 8.0). Presence of purified protein from each fraction was probed via Bradford test and SDS‐PAGE, and fractions containing CpxR‐6xHis were combined. Imidazole was removed via three rounds of dialysis in 4 L of Elution Buffer without imidazole and 2 mM DTT. The purified protein was then concentrated using a 3000 NMWL concentrator spun at 3000 rcf at 4°C. Purified protein was stored in the presence of 10% glycerol at −70°C.

### 4.11 Electrophoretic Mobility Shift Assay (EMSA)

Plasmid templates and primers listed in Tables S2 and S3, respectively, were used for PCR amplification of the *yscW*‐*lcrF* intergenic region and the promoter regions of *cpxR*, *yscW*‐*lcrF*, *ymoBA*, and *E. coli sodA*. The amplified DNA fragments were purified using the QIAquick PCR purification kit (Qiagen). DNA fragments (∼4–8 nM) were incubated with or without purified CpxR in a 10 μL reaction volume containing 20 mM Tris–HCl pH 7.9, 125 mM KCl, 10 mM MgCl_2_, 1 mM EDTA, 1 mM DTT, and 100 μg mL^−1^ bovine serum albumin. Where indicated, the reaction mixture also contained 30 mM acetyl phosphate. After incubation at 30°C for 1 h, heparin was added to 50 μg ml^−1^, and the mixture was further incubated at 30°C for 2 min. Glycerol was added to 10% before samples were loaded onto a nondenaturing 6% polyacrylamide gel. Gel electrophoresis was performed in 0.5X Tris‐borate‐EDTA (TBE) buffer at 200 V for 4–5 h at 4°C. The gel was stained with SYBR green EMSA nucleic acid gel stain (Molecular Probes) and visualized using a Typhoon FLA 900 imager (GE).

## Author Contributions

**Patricia J. Kiley:** funding acquisition, writing – review and editing, supervision. **Erin Mettert:** investigation, writing – review and editing, methodology. **Karen Hug:** investigation, writing – original draft, methodology, validation, visualization, writing – review and editing, formal analysis, data curation, conceptualization. **Sarvind Tripathi:** resources. **David Balderas:** conceptualization, investigation. **Cristina Vargas Vazquez:** investigation. **Melissa Estrada Escobar:** investigation. **Laya Ashley:** investigation. **Victoria Auerbuch:** supervision, formal analysis, project administration, data curation, visualization, writing – review and editing, writing – original draft, funding acquisition, conceptualization.

## Funding

This work was supported by National Institutes of Health, R01AI119082, R01AI187307, 4R25HG006836, 5R25GM104552.

## Disclosure

All data, figures, tables, and text in this manuscript are original and were created by the authors. No copyrighted, trademarked, or previously published material requiring permission has been used in this work. The graphical abstract was made using BioRender.

## Ethics Statement

The authors have nothing to report.

## Conflicts of Interest

The authors declare no conflicts of interest.

## Supporting information


**Data S1:** mmi70076‐sup‐0001‐DataS1.xlsx.

**Figure S1:** Effect of IPTG addition on secretion in wildtype and Δ*cpxR* *Y. pseudotuberculosis* harboring pTRC99a empty vector.
**Figure S2:** Phosphorylated CpxR does not bind the *yscW‐lcrF* promoter region.
**Figure S3:** Representative image of YmoA western blot time‐course.
**Figure S4:** Select CpxR‐regulated genes were deleted in *Y. pseudotuberculosis* , and LcrF levels were assessed.
**Figure S5:** The *cpxR* ^D51E^ *∆ompR* mutant displays a growth defect.
**Figure S6:** Normalized RNA‐Seq reads for OmpR‐regulated *ompF* and *ompC* genes.
**Figure S7:** CpxR activation does not alter *rcsB* mRNA levels.
**Figure S8:** Serotonin does not influence LcrF expression.
**Table S1:** *Yersinia pseudotuberculosis* strains used in this study.
**Table S2:** Plasmids used in this study.
**Table S3:** Primers used in this study.
**Dataset: S1** Genes differentially expressed in *∆cpxA* and *cpxR* ^D51E^ mutant strains compared to wildtype *Y. pseudotuberculosis* .

## Data Availability

The data that support the findings of this study are openly available in the NCBI Gene Expression Omnibus at https://www.ncbi.nlm.nih.gov/geo/, reference number GSE313348.
