# Supplementary material for: Regulation of the Yersinia pseudotuberculosis Type III Secretion System by the CpxAR Two‐Component System
Source: Mol Microbiol. 2026 May 24;126(1):81–97. doi: 10.1111/mmi.70076 (PMC13353760; doi:10.1111/mmi.70076)
Supplement: Supplementary file 2 — Figure S1: Effect of IPTG addition on secretion in wildtype and ΔcpxR Y. pseudotuberculosis harboring pTRC99a empty vector. Figure S2: Phosphorylated CpxR does not bind the yscW‐lcrF promoter region. Figure S3: Representative image of YmoA western blot time‐course. Figure S4: Select CpxR‐regulated genes were deleted in Y. pseudotuberculosis , and LcrF levels were assessed. Figure S5: The cpxR D51E ∆ompR mutant displays a growth defect. Figure S6: Normalized RNA‐Seq reads for OmpR‐regulated ompF and ompC genes. Figure S7: CpxR activation does not alter rcsB mRNA levels. Figure S8: Serotonin does not influence LcrF expression. Table S1: Yersinia pseudotuberculosis strains used in this study. Table S2: Plasmids used in this study. Table S3: Primers used in this study. Dataset: S1 Genes differentially expressed in ∆cpxA and cpxR D51E mutant strains compared to wildtype Y. pseudotuberculosis . [file MMI-126-81-s001.pdf]

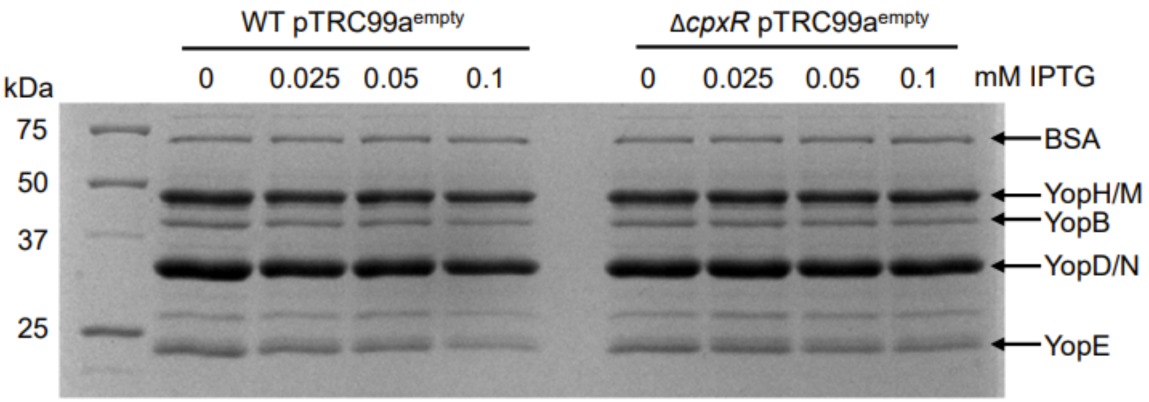

Figure S1. Effect of IPTG addition on secretion in wildtype and Δ*cpxR Y. pseudotuberculosis* harboring pTRC99a empty vector. Wildtype and Δ*cpxR Y. pseudotuberculosis* strains harboring a pTRC99a empty vector were grown in T3SS-inducing conditions in the presence of varying concentrations of IPTG. A representative gel out of three independent experiments is shown.

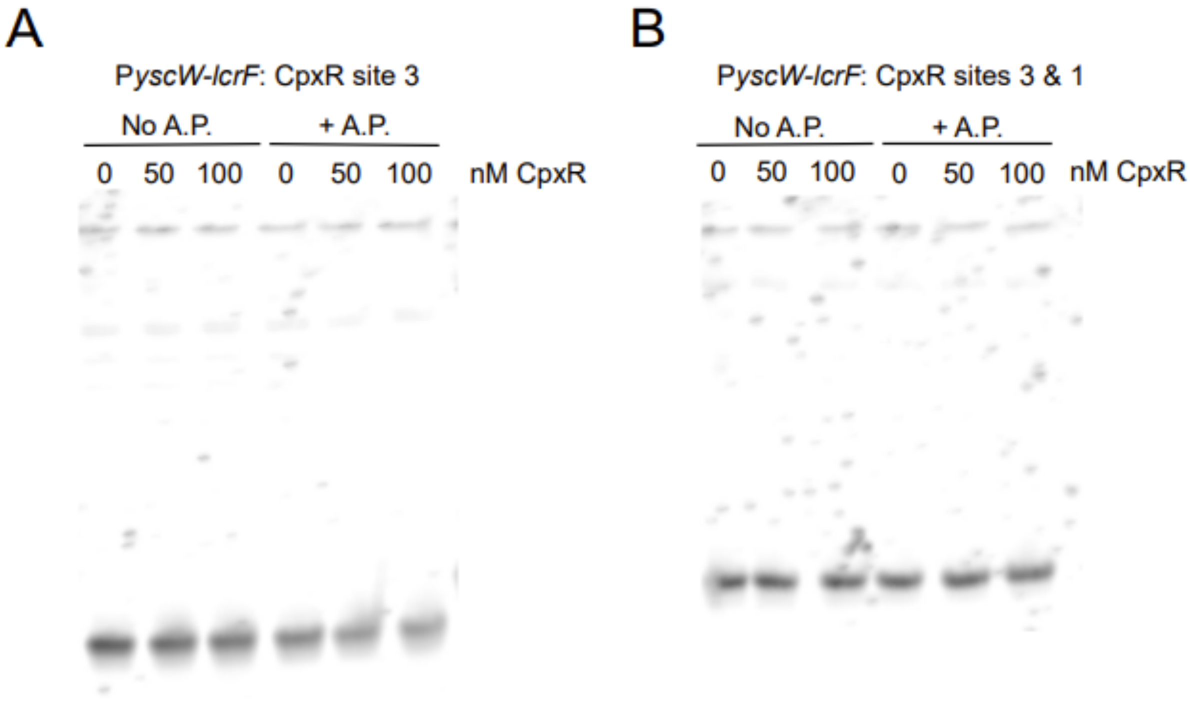

Figure S2. Phosphorylated CpxR does not bind the *yscW-lcrF* promoter region. Purified CpxR incubated with and without acetyl phosphate (A.P.) was used for EMSA analysis using a fragment of the *Yersinia yscW-lcrF* promoter containing **(A)** only site 3 (p = 0.00193, -379 to -179 bp relative to the TSS, generated with primers pPK7179_P*yscW*_F1 and pPK7179_P*yscW*_R2) and **(B)** site 1 (p = 0.00132) and site 3 (p = 0.00193) (-313 to -129 bp relative to the TSS, generated with primers M13R and pPK7179_P*yscW*_R3).

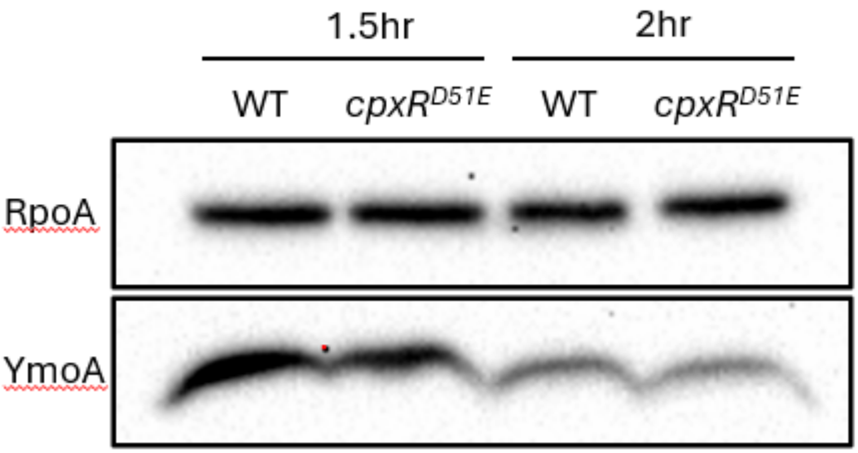

Figure S3. Representative image of YmoA Western blot time-course. Wildtype and *cpxR*^{D51E} *Y. pseudotuberculosis* were grown in low calcium LB at 26˚C for 1.5 hours before being shifted to 37˚C to induce the T3SS. Samples were taken at 1.5 hours post-shift to 37˚C as well as 2 hours post-shift to account for potential delays between transcription and translation, as *ymoA* mRNA levels were seen to be elevated at 1.5 hours. YmoA protein levels were assayed via western blot.

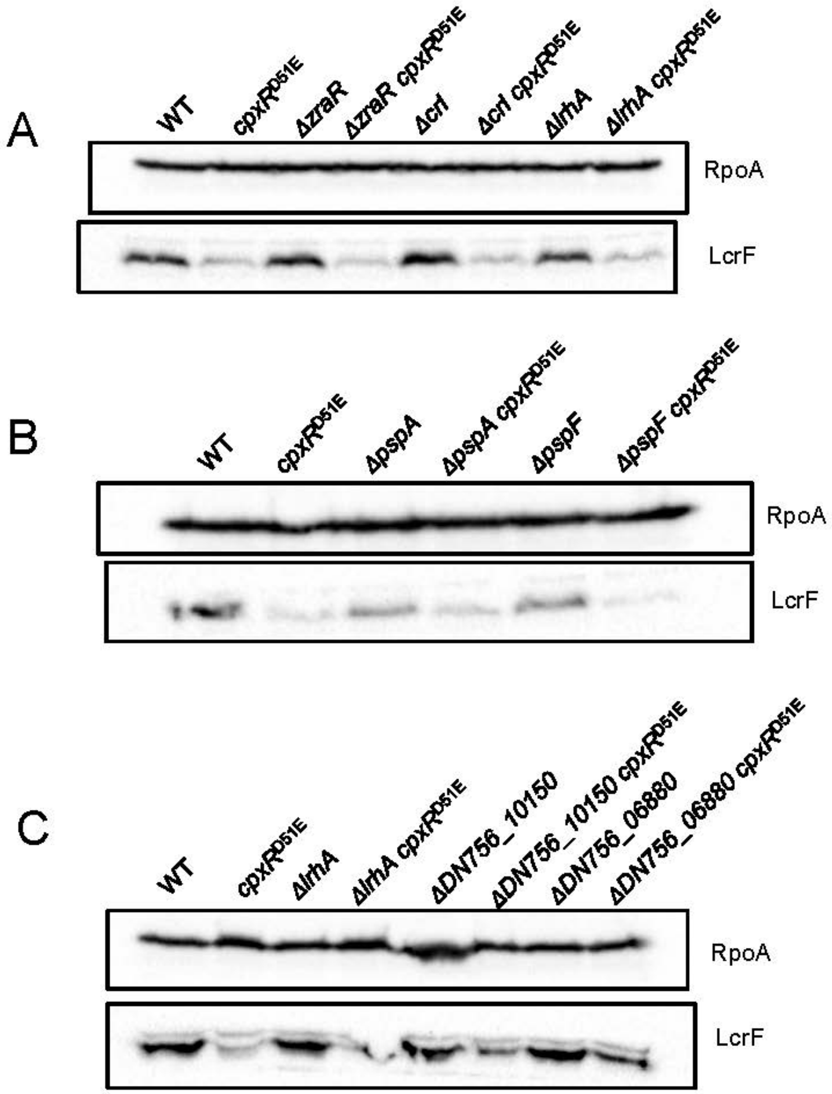

Figure S4. Select CpxR-regulated genes were deleted in *Y. pseudotuberculosis*, and LcrF levels were assessed. *Y. pseudotuberculosis* strains were grown under T3SS-inducing conditions and LcrF protein levels relative to RpoA were assessed via western blots. Densitometry was used to measure the relative amount of LcrF relative to the RpoA control. Representative blots out of three independent experiments are shown for all strains excepting Δ*crl* and Δ*zraR* strains, which are representative of two independent experiments.

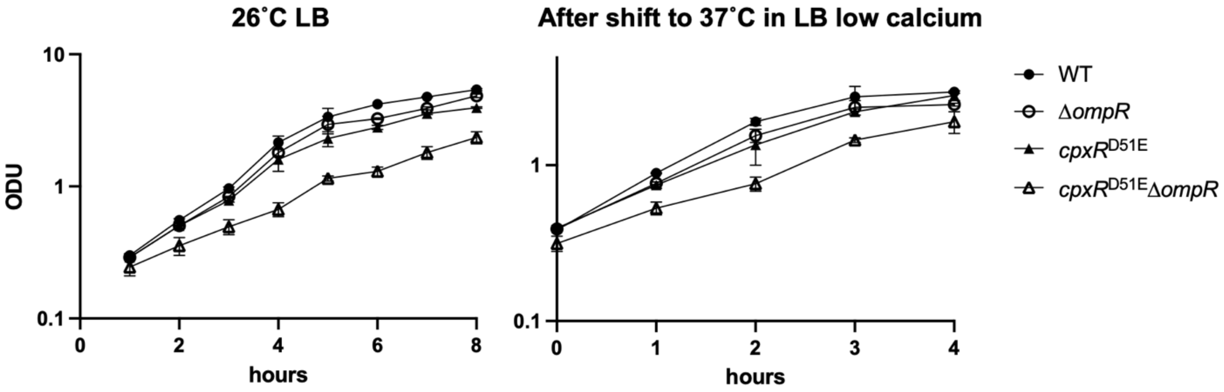

Fig S5. The *cpxR*^{D51E}Δ*ompR* mutant displays a growth defect. *Y. pseudotuberculosis* strains were grown in LB medium at 26˚C for a total of 8 hours. OD_{600} was recorded every hour to track growth. Error bars that are not visible are smaller than the symbol for that data point. The same strains were grown in low calcium LB at 26˚C for 1.5 hours and then shifted to 37˚C for a total of 4 hours. OD_{600} was recorded before the shift to 37˚C (time 0) and each hour following. Average ± standard error of the mean of two independent experiments is shown.

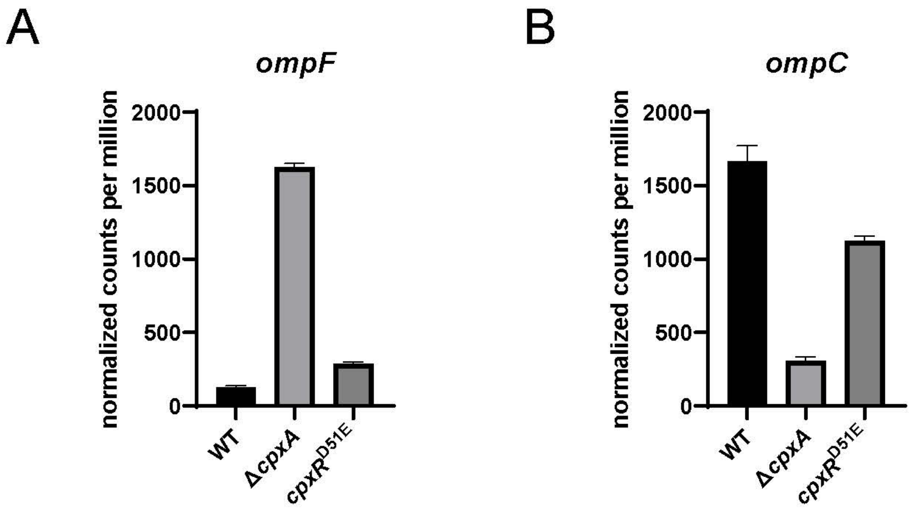

Figure S6. Normalized RNA-Seq reads for OmpR-regulated *ompF* and *ompC* genes.

Average TMM values ± StDev are shown for three independent experiments. RNA-seq analysis determined that *ompF* was differentially expressed in both the Δ*cpxA* and *cpxR*^{D51E} mutants **(A)** and that *ompC* was differentially expressed in the Δ*cpxA* mutant **(B).**

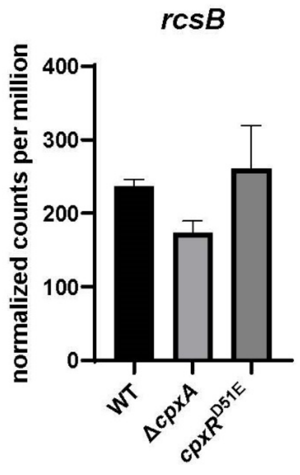

Figure S7. CpxR activation does not alter *rcsB* mRNA levels. Average TMM values from the RNA-Seq analysis ± StDev are shown for three independent experiments.

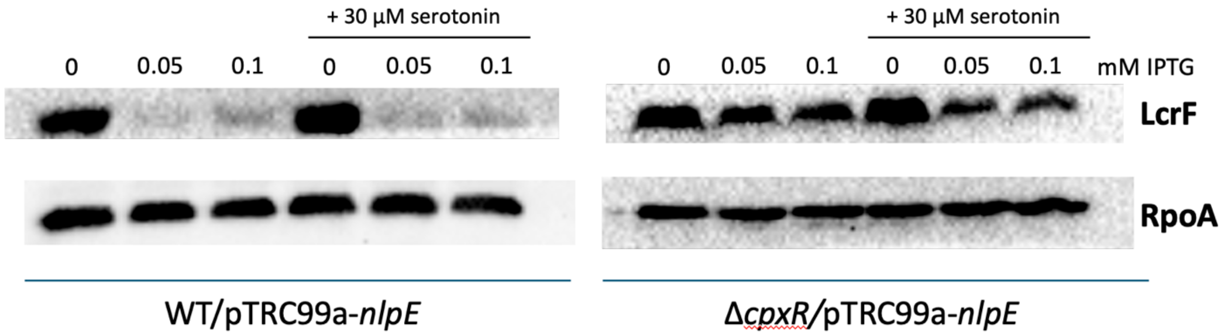

Figure S8. Serotonin does not influence LcrF expression. Wildtype and Δ*cpxR Y. pseudotuberculosis* strains harboring a pTRC99a-*nlpE* overexpression vector were grown in T3SS-inducing conditions in the presence of varying concentrations of IPTG and in the presence or absence of 30 µM serotonin. LcrF and RpoA pellet levels were assessed via western blot. A representative of two independent experiments is shown.

Table S1: ***Yersinia pseudotuberculosis*** **strains used in this study**

Strain	Description	Source
Wildtype IP2666	*Y. pseudotuberculosis* IP2666, naturally lacks YopT	(Bliska et al., 1991)
Δ*cpxA*	*cpxA* in-frame deletion of codons 41 to 449	(Liu et al., 2012)
cpxR^{D51E}	*cpxR* with aspartate to glutamate mutation at residue 51	(Thanikkal et al., 2012)
Δ*ymoA*	Full in-frame deletion of *ymoA*	(Böhme et al., 2012)
Δ*ymoA* Δ*cpxA*	*cpxA* in-frame deletion of codons 41 to 449 in Δ*ymoA* background	This work
Δ*ymoA* *cpxR*^{D51E}	*cpxR* with aspartate to glutamate mutation at residue 51 in Δ*ymoA* background	This work
Δ*cpxR*	Full in-frame deletion of cpxR	This work
Δ*cpxR* Δ*cpxA*	Double deletion of *cpxR* and *cpxA*	This work
Δ*pspA*	Clean in-frame deletion of *pspA*	This work
Δ*pspF*	Clean in-frame deletion of *pspF*	This work
Δ*mzrA*	Deletion of *mzrA*	This work
Δ*zraR*	Deletion of *zraR*	This work
Δ*lrhA*	Clean in-frame deletion of *lrhA*	This work
Δ*crl*	Clean in-frame deletion of *crl*	This work
ΔDN756_10150	Clean in-frame deletion of DN756_10150	This work
ΔDN756_06880	Clean in-frame deletion of DN756_06880	This work
cpxR^{D51E} Δ*pspA*	Clean in-frame deletion of *pspA* in *cpxR*^{D51E} background	This work
cpxR^{D51E} Δ*pspF*	Clean in-frame deletion of *pspF* in *cpxR*^{D51E} background	This work
cpxR^{D51E} Δ*mzrA*	Deletion of *mzrA* in *cpxR*^{D51E} background	This work
cpxR^{D51E} Δ*zraR*	Deletion of *zraR* in *cpxR*^{D51E} background	This work
cpxR^{D51E} Δ*lrhA*	Clean in-frame deletion of *lrhA* in *cpxR*^{D51E} background	This work
cpxR^{D51E} Δ*crl*	Clean in-frame deletion of crl in *cpxR*^{D51E} background	This work
cpxR^{D51E}ΔDN756_10150	Clean in-frame deletion of DN756_10150 in *cpxR*^{D51E} background	This work

cpxR^{D51E}ΔDN756_06880	Clean in-frame deletion of DN756_06880 in *cpxR*^{D51E} background	This work
Δ*ompR*	Clean in-frame deletion of *ompR*	This work
cpxR^{D51E} Δ*ompR*	Clean in-frame deletion of *ompR* in *cpxR*^{D51E} background	This work
Δ*lcrF*	In-frame deletion of *lcrF*	(Garrity-Ryan et al., 2010)

Table S2: Plasmids used in this study

Plasmid	Description	Source
pSR47S Δ*cpxA*	Suicide vector for *cpxA* deletion, Kan^R	This work
pSR47S Δ*cpxR*	Suicide vector for *cpxR* deletion, Kan^R	This work
pSR47S CpxR^{D51E}	Suicide vector containing 500bp surrounding D51E mutation site, Kan^R	This work
pSR47S Δ*ymoA*	Suicide vector for *ymoA* deletion, Kan^R	This work
pTRC99a-*nlpE*	IPTG-inducible *nlpE* overexpression vector, Carb^R	This work
pFU99a-*pymoBA*::*lacZ*	ymoBA promoter fused to lacZ, Cm^R	This work
pSR47S Δ*pspA*	Suicide vector for *pspA* deletion, Kan^R	This work
pSR47S Δ*pspF*	Suicide vector for *pspF* deletion, Kan^R	This work
pSR47S Δ*lrhA*	Suicide vector for *lrhA* deletion, Kan^R	This work
pSR47S Δ*mzrA*	Suicide vector for *mzrA* deletion, Kan^R	This work
pSR47S Δ*crl*	Suicide vector for *crl* deletion	This work
pSR47S Δ*zraR*	Suicide vector for *zraR* deletion, Kan^R	This work
pSR47S ΔDN756_06880	Suicide vector for DN756_06880 deletion, Kan^R	This work
pSR47S ΔDN756_10150	Suicide vector for DN756_10150 deletion, Kan^R	This work
pSR47S Δ*ompR*	Suicide vector for *ompR* deletion, Kan^R	This work
pFU99a-*pyscW::lacZ*	*yscW-lcrF* promoter construct 2 fused to lacZ, Cm^R	(Balderas et al., 2022)
pPK7179_*lcrF*_intergenic	Vector containing EMSA fragment of *yscWlcrF* intergenic region, Amp^R	This work
pPK7179_P*cpxR*	Vector containing EMSA fragment of *cpxR* promoter region positive control, Amp^R	This work
pPK7179_P*yscWlcrF*_short	Vector containing 575bp EMSA fragment of between *yscU* stop codon and *yscW* start codon from which	This work

	smaller fragments were assayed, Amp^R	
pPK7179_P*yscWlcrF*_long	Vector containing -511 to +263 bp relative to the TSS from which smaller fragments were assayed for EMSA, Amp^R	This work
pPK7179_P***ymoBA***	Vector containing 200 bp fragment upstream of *ymoB* start codon for EMSA	This work
pET28b CpxR-His	pET28, CpxR-6xHis, Kan^R	This work
**pPK6869**	Vector containing EMSA fragment of *E. coli sodA* promoter region negative control, Amp^R	Giel et al., 2006

Table S3: Primers used in this study

Name	Primer sequence	Source
qPCR_*16s*_F	AGCCAGCGGACCACATAAAG	(Yang et al., 1996)
qPCR_*16s*_R	AGTTGCAGACTCCAATCCGG	(Yang et al., 1996)
qPCR_*cpxP*_F	CGGTGACGGTAAGATGATGATG	This work
qPCR_*cpxP*_R	CGCATCAAGTCACGCATTTG	This work
F_pET28bCpxRHis	tttttCCATGGCCATGCATAATCCTATTAGT	This work
R_pET28bCpxRHis	tttttCTCGAGTGTTTCTGATACCATCAAGTAG	This work
qPCR_*ymoA*_F	CCTGATGCGTTTAAGAAAATG	(Balderas et al., 2022)
qPCR_*ymoA*_R	GATGGTCTGCAGCTGAGTAAA	(Balderas et al., 2022)
F5'Δ*cpxA*	cgaattcctgcagcccggggAATGGAGGGTTTCAATGTTG	This work
R5'Δ*cpxA*	gcggtagccaGTCGAGTAAAACAGTGAG	This work
F3'Δ*cpxA*	tttactcgacTGGCTACCGCTGCATCCG	This work
R3'Δ*cpxA*	agggaacaaaagctggagctTGCACAACTGTCTCATGGCG	This work
F5'Δ*ymoA*	cgaattcctgcagcccggggGATAGACAGCTGTATTTATATGAC	(Balderas et al., 2022)
R5'Δ*ymoA*	gcgctaagcaGGTTTTTCTTCTCGATATACAAATTAATATTG	(Balderas et al., 2022)

F3'Δ*ymoA*	aagaaaaaccTGCTTAGCGCTGGTTAAG	(Balderas et al., 2022)
R3'Δ*ymoA*	agggaacaaaagctggagctCCTGTATTATCACTTTCCTGC	(Balderas et al., 2022)
F_pSR47s_ CpxR^{D51}	AGGGAACAAAAGCTGGAGCTAGTGAGGTTGATGCCATCAAAC	This work
R_pSR47s_ CpxR^{D51}	CGAATTCCTGCAGCCCGGGGAAACCACGGTAGCCCGTC	This work
5' mutagenesis CpxR^{D51E}	GTTATTGCTTGAAATTATGATGC	This work
3' mutagenesis CpxR^{D51E}	AAGTCGATAGAGCTATCTAG	This work
F5'Δ*cpxR*	cgaattcctgcagcccggggCCATTTTATGATGAATAGCAG	This work
R5'Δ*cpxR*	ctgtttatcaCATGGTTATTTCTCCTCTC	This work
F3'Δ*cpxR*	aataaccatgTGATAAACAGTTTAACGACG	This work
R3'Δ*cpxR*	agggaacaaaagctggagctGTGACTACCAGCGTTTTAC	This work
F_pTRC99a_*nlpE*	ATTTCACACAGGAAACAGACATGACTTCATTCCATATATATCG	This work
R_pTRC99a_*nlpE*	TGCATGCCTGCAGGTCGACTCTATTTTTCTCACAACTTTTATTG	This work
pFU99_*PymoBA*_F	cctttcgtcttcacctcgagTAATTGGTATATTTTCAATGCTTGTTTGGATATCAATAC	(Balderas et al., 2022)
pFU99_*PymoBA*_R	ttcatttttaattcctcctgGTCATGCCGCTTAGGCGAG	(Balderas et al., 2022)
pFU99_*PyscW*_F	cctttcgtcttcacctcgagAGGCTGCAATGTAACTAG	(Balderas et al., 2022)
pFU99_*PyscW*_R	ttcatttttaattcctcctgAGAAATGATGAGTGCTATAATACG	(Balderas et al., 2022)

pspA US F	AGGGAACAAAAGCTGGAGCTAGGCCCCGGCTTCATGG	This work
pspA US R	AAAATTAACTACATAATTTACGTCCCCTTTGACTTATTGG	This work
pspA DS F	GTAAATTATGTAGTTAATTTTCCGTATTTATTAGC	This work
pspA DS R	CGAATTCCTGCAGCCCGGGGCCAACCGTACTGGAATATC	This work
pspF US F	AGGGAACAAAAGCTGGAGCTCTTCTTTATCTTTACGCAAG	This work
pspF US R	GCTGTACTCACATGATGAAATTCGCCAATAG	This work
pspF DS F	TTTCATCATGTGAGTACAGCTAATTTTCATTG	This work
pspF DS R	CGAATTCCTGCAGCCCGGGGATCCATTGCACAAAACAAC	This work
mzrA US F	AGGGAACAAAAGCTGGAGCTGTTTTCCAGTGACCCTGG	This work
mzrA US R	GCGTTGATTATCACTCTGACTTACCTTTCTTATTG	This work
mzrA DS F	GTCAGAGTGATAATCAACGCTACGGGGAG	This work
mzrA DS R	CGAATTCCTGCAGCCCGGGGTCTTGTTGGCGCTCTGTCA	This work
zraR US F	AGGGAACAAAAGCTGGAGCTTTTAAGGATCCAACTCGTC	This work
zraR US R	AAAATAGCTATTATGTGTTGTCATTATTCGAC	This work
zraR DS F	CAACACATAATAGCTATTTTTTTATAAAAATACAGGCATTTTAAAAAG	This work
zraR DS R	CGAATTCCTGCAGCCCGGGGTAGATGCCGGGCGTAAAG	This work
lrhA US F	AGGGAACAAAAGCTGGAGCTATCATCATACATATTGGCTG	This work

lrhA US R	AGATTACTTACATAGTGTATTCTTCACTTTTTTTG	This work
lrhA DS F	ATACACTATGTAAGTAATCTGTTTTTAATTCATCATC	This work
lrhA DS R	CGAATTCCTGCAGCCCGGGGAGTAACTACACATTAATTTACAAAC	This work
crl US F	AGGGAACAAAAGCTGGAGCTTTCGGTTTTCGTTTTGGG	This work
crl US R	CGTATTATCACATACTTAAATCTCCTTATGTAGAC	This work
crl DS F	TTTAAGTATGTGATAATACGCTTACCTAGGTTTGATTAG	This work
crl DS R	CGAATTCCTGCAGCCCGGGGATCTGCGCGGGTTAGCAG	This work
DN756_10150 US F	AGGGAACAAAAGCTGGAGCTCATTGGAGAAGCGAATGTCTG	This work
DN756_10150 US R	AATAAGACTACATAGCTTTCATCTCCGCATTAAATC	This work
DN756_10150 DS F	GAAAGCTATGTAGTCTTATTAAACAGCCCAC	This work
DN756_10150 DS R	CGAATTCCTGCAGCCCGGGGAGACATAGATAAAAGTTAGATTTTG	This work
DN756_06880 US F	AGGGAACAAAAGCTGGAGCTTCAGCGCATTTTGTTGAG	This work
DN756_06880 US R	GTGACGGTTACATTTTTAACTCCCCTACC	This work
DN756_06880 DS F	GTTAAAAATGTAACCGTCACTGCGGAGTG	This work
DN756_06880 DS R	CGAATTCCTGCAGCCCGGGGTCCAGATCATTGGGATTGGAAC	This work
ompR US F	AGGGAACAAAAGCTGGAGCTGAAAGAAGCGGCTGAGAG	This work
ompR US R	CACTTCATGCCATTTTATTACTCCCAAAGGC	This work
ompR DS F	TAATAAAATGGCATGAAGTGGTGGCGCTTTTC	This work

ompR DS R	CGAATTCCTGCAGCCCGGGGAATGGCCAAGGTATAACGGAAC	This work
pPK7179_lcrF_IG_F	CAAGCTTGCATGCCTGCAGCTCGAGTCCAGAGCGAGGAGTTCA	(Liu et al., 2012)
pPK7179_lcrF_IG_R	GAACATCTTACCTCTGTGGGGATCCTATAGGCCATCTTGTGAATG	(Liu et al., 2012)
pPK7179 PcpxR_F	CAAGCTTGCATGCCTGCAGCTCGAGCGTTAACTCCTAAAGTTCAATATG	This work
pPK7179 PcpxR_R	GAACATCTTACCTCTGTGGGGATCCGGTTATTTCTCCTCTCTG	This work
M13R	CAGGAAACAGCTATGAC	This work
pPK7179_BamHI downstream R	GGTCTGAAAGATAGAAC	This work
sodA-1	TCACTCGAGCATCTCCGACGAGATGA	Giel et al., 2006
sodA-2	TAGGATCCATTGTCGGGCGGCCGATT	Giel et al., 2006
pPK7179_PyscW_R1	AAATGCACAGGAGAAATACAATTACC	This work
pPK7179_PyscW_F1	ACAGCTGAAGTGCTACGATGGCTAG	This work
pPK7179_PyscW_R2	ATTACATATTCCCAATAGCCGGTGTT	This work
pPK7179_PyscW_R3	ATATATTCCTAATATAAGTGAACCTCTTGTTG	This work

Dataset S1. Genes differentially expressed in Δ*cpxA* and *cpxR*$^{\mathrm{D51E}}$ mutant strains compared to wildtype *Y. pseudotuberculosis*.

Supplemental References

Bliska, J.B., Guan, K.L., Dixon, J.E., Falkow, S., 1991. Tyrosine phosphate hydrolysis of host proteins by an essential *Yersinia* virulence determinant. Proceedings of the National Academy of Sciences 88, 1187–1191. https://doi.org/10.1073/pnas.88.4.1187

Garrity-Ryan, L.K., Kim, O.K., Balada-Llasat, J.-M., Bartlett, V.J., Verma, A.K., Fisher, M.L., Castillo, C., Songsungthong, W., Tanaka, S.K., Levy, S.B., Mecsas, J., Alekshun, M.N., 2010. Small Molecule Inhibitors of LcrF, a *Yersinia pseudotuberculosis* Transcription Factor, Attenuate Virulence and Limit Infection in a Murine Pneumonia Model. Infection and Immunity 78, 4683–4690. https://doi.org/10.1128/iai.01305-09

Giel, J.L., Rodionov, D., Liu, M., Blattner, F.R., Kiley, P.J., 2006. IscR-dependent gene expression links iron-sulphur cluster assembly to the control of O_2-regulated genes in *Escherichia coli*. Mol Microbiol 60, 1058–1075. https://doi.org/10.1111/j.1365-2958.2006.05160.x

Yang, Y., Merriam, J.J., Mueller, J.P., Isberg, R.R., 1996. The psa locus is responsible for thermoinducible binding of *Yersinia pseudotuberculosis* to cultured cells. Infection and Immunity 64, 2483–2489. https://doi.org/10.1128/iai.64.7.2483-2489
